# Supplementary material for: A SAM-I riboswitch with the ability to sense and respond to uncharged initiator tRNA
Source: Nat Commun. 2020 Jun 3;11:2794. doi: 10.1038/s41467-020-16417-z (PMC7270179; doi:10.1038/s41467-020-16417-z)
Supplement: Supplementary file 1 — Supplementary Information [file 41467_2020_16417_MOESM1_ESM.pdf]

Supplementary Information

SAM-I riboswitch with the ability to sense and respond to uncharged initiator tRNA.
By Tang *et al*.

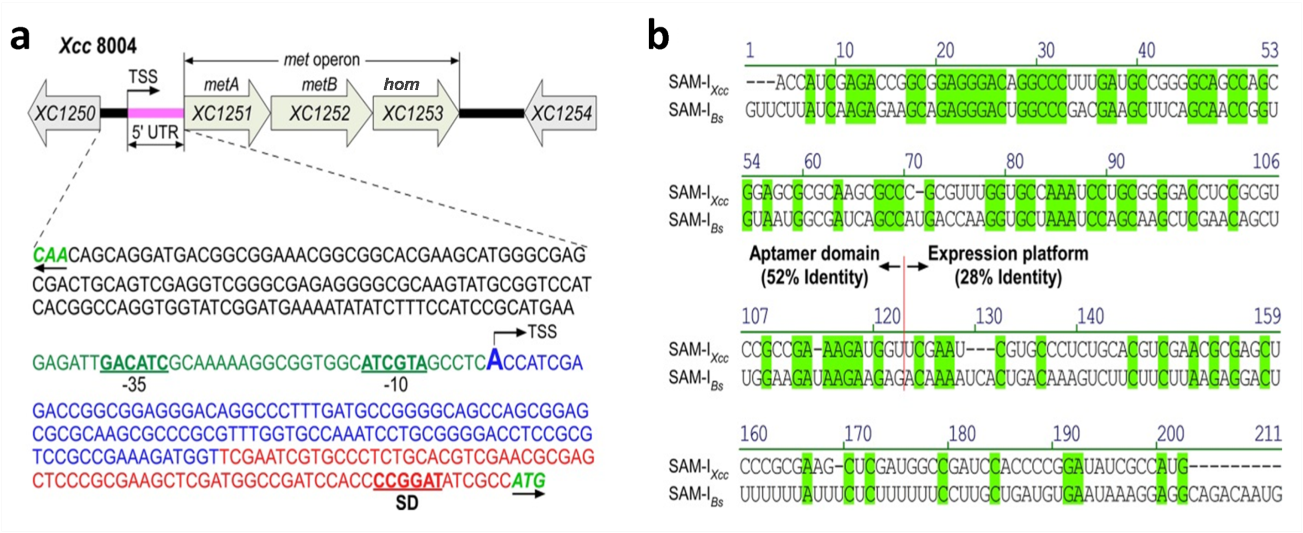

SupplementaryFig. 1 | SAM-I*Xcc* riboswitch in *Xcc*. (a) The location and DNA sequence of SAM-I*Xcc*. Description of the genetic organization can be found in Fig. 1. The blue and red letters denote the aptamer-coding and expression platform-coding sequences, respectively. The two translational start codons of *XC1250* and *XC1251* are marked in light green and indicated by arrows. Underlined bold letters in dark green indicate the -10 and -35 region of the promoter of the *met* operon, and underlined bold letters in red indicate the Shine-Dalgarno (SD) sequence of the *met* mRNA. **(b)** Alignment of the sequences of SAM-I*Xcc* and the *yitJ* SAM-I riboswitch of *Bacillus subtilis* (SAM-I*Bs*)[14]. Letters with green shadow denote the identical nucleotides.

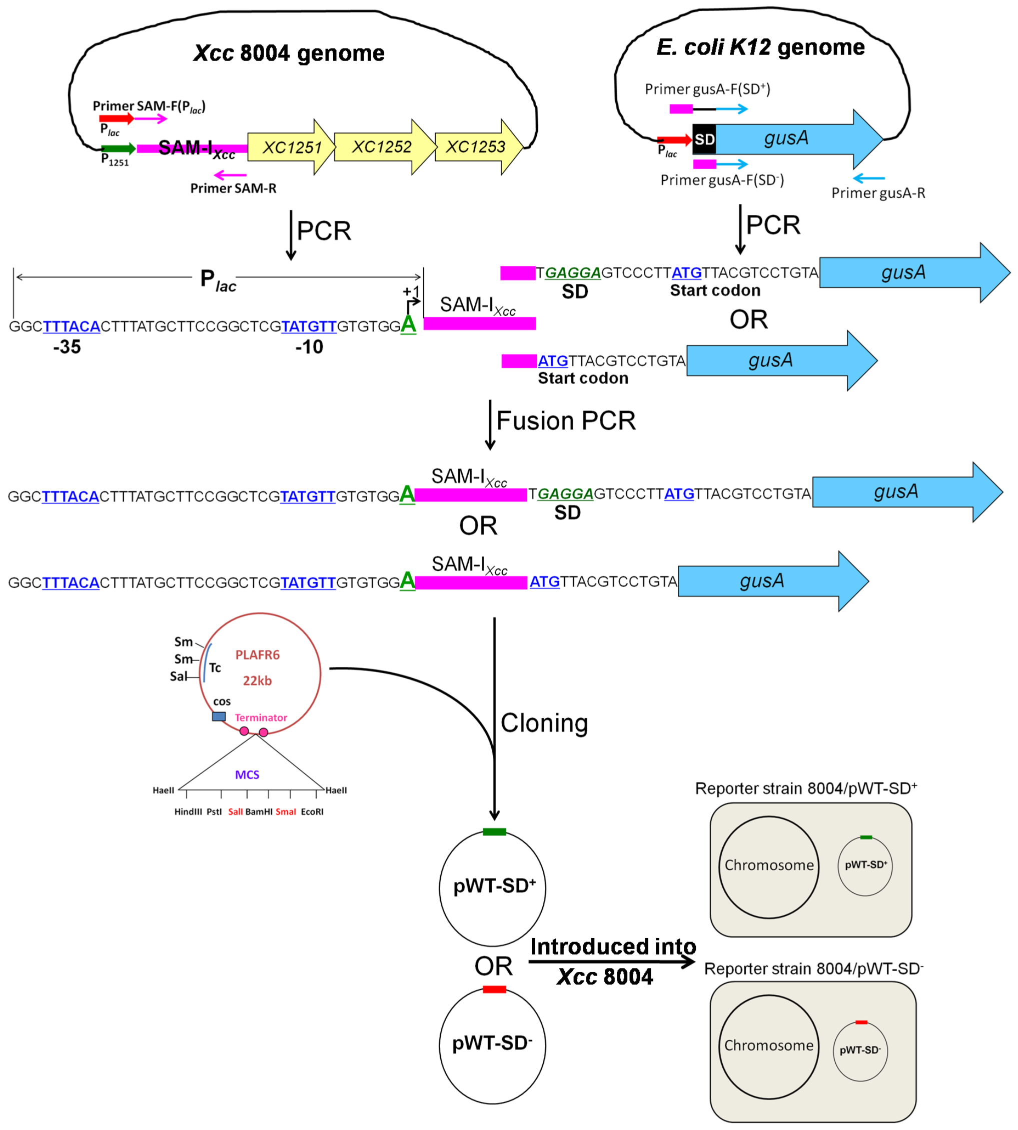

Supplementary Fig. 2 | Diagram describing the construction of SAM-I*Xcc*-*gusA* reporter. A DNA fragment containing the SAM-I*Xcc* coding sequence with the *lac* promoter was obtained by PCR amplification using the *Xcc* 8004 DNA as template. Meanwhile, a DNA fragment containing the *gusA*-coding sequence was generated by PCR amplification using the *E.coli* K12 genomic DNA as template. The two DNA fragments were then fused into one by fusion PCR[47], which was cloned into the vector pLAFR6. The resulting reporter plasmid was introduced into *Xcc* strain 8004 to create a reporter strain. Reporters with mutations in SAM-I*Xcc* were constructed in a similar way.

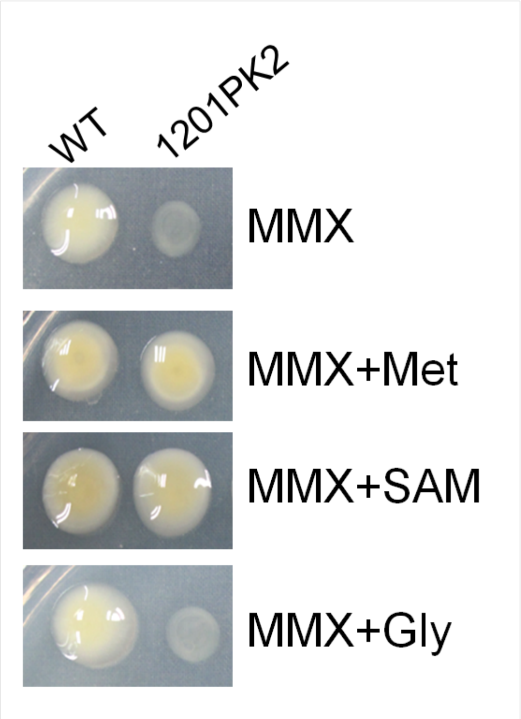

Supplementary Fig. 3 |Growth of *met* operon mutant in methionine-deficient medium with addition of SAM. Plate assay for detection of the growth of the wilt type strain *Xcc* strain 8004 (WT) and the *met* operon inactivation mutant 1201PK2[26] in the minimal medium MMX, and MMX supplemented with 1mM of methionine (MMX+Met), SAM (MMX+SAM) or glycine (MMX+Gly). About 2 μl overnight culture (OD_{600} ≈ 1.0) of each strain (in the rich medium NYGB) was respectively spotted onto the MMX agar plates, and results were observed and photographed after incubation at 28 ºC for3 days. Source data are provided as a Source Data file.

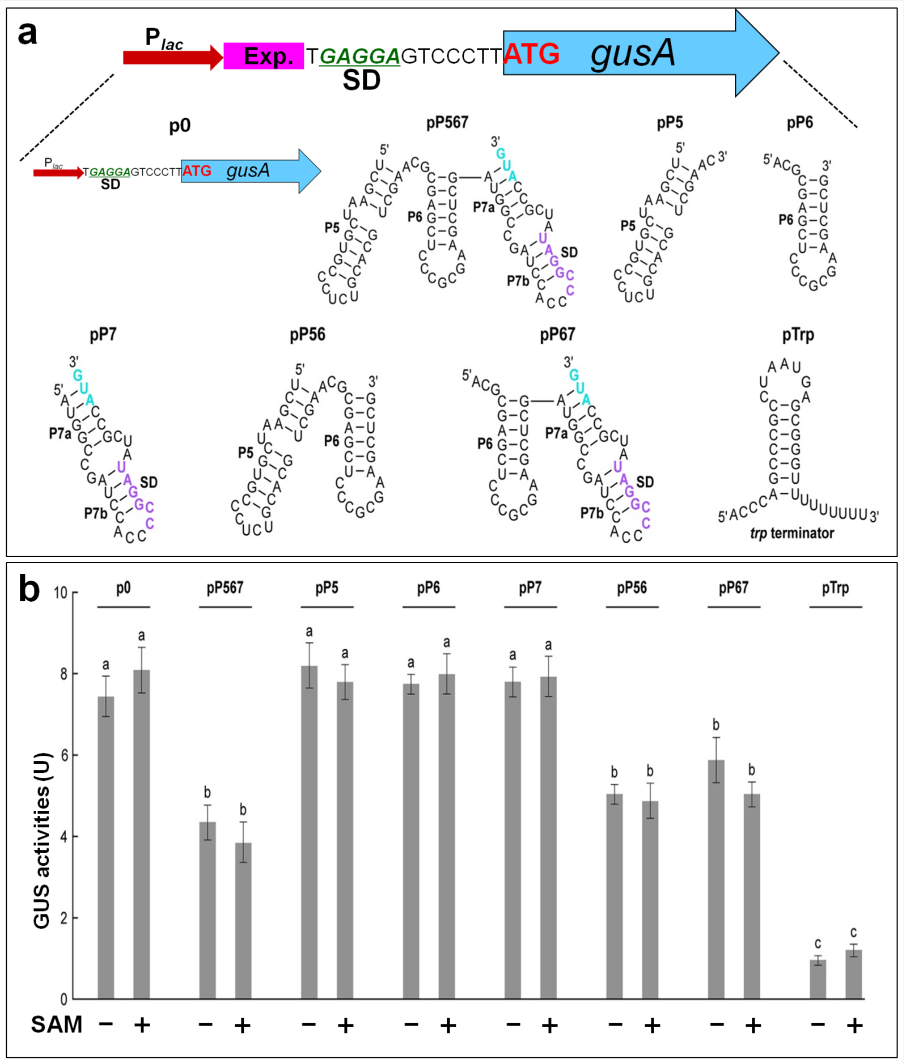

Supplementary Fig. 4 |Investigation of the role of the three hairpin structures in the expression platform of SAM-I$_{Xcc}$ by *gusA* transcriptional fusion reporter construction and GUS activity assay. (a) The genetic organization of the *gusA* transcriptional reporters. The construct without SAM-I$_{Xcc}$ expression platform sequence insertion between the *lac* promoter (P$_{lac}$) and the SD+*gusA* was designated as p0, the construct with an insertion of the full-length or truncated expression platform was designated as pP567, pP5, pP6, pP7, pP56, and pP67, respectively, and the construct with an insertion of a *trp* terminator was designated as pTrp. **(b)** the GUS activities produced by the corresponding reporter strains in the presence (300 µM) (SAM+) and absence of SAM (SAM-). Data are presented as mean values ± SD from three biologically independent samples. Significant differences between GUS activities at $P < 0.01$ (Student's two-tailed *t*-test) are marked by different letters. Source data are provided as a Source Data file.

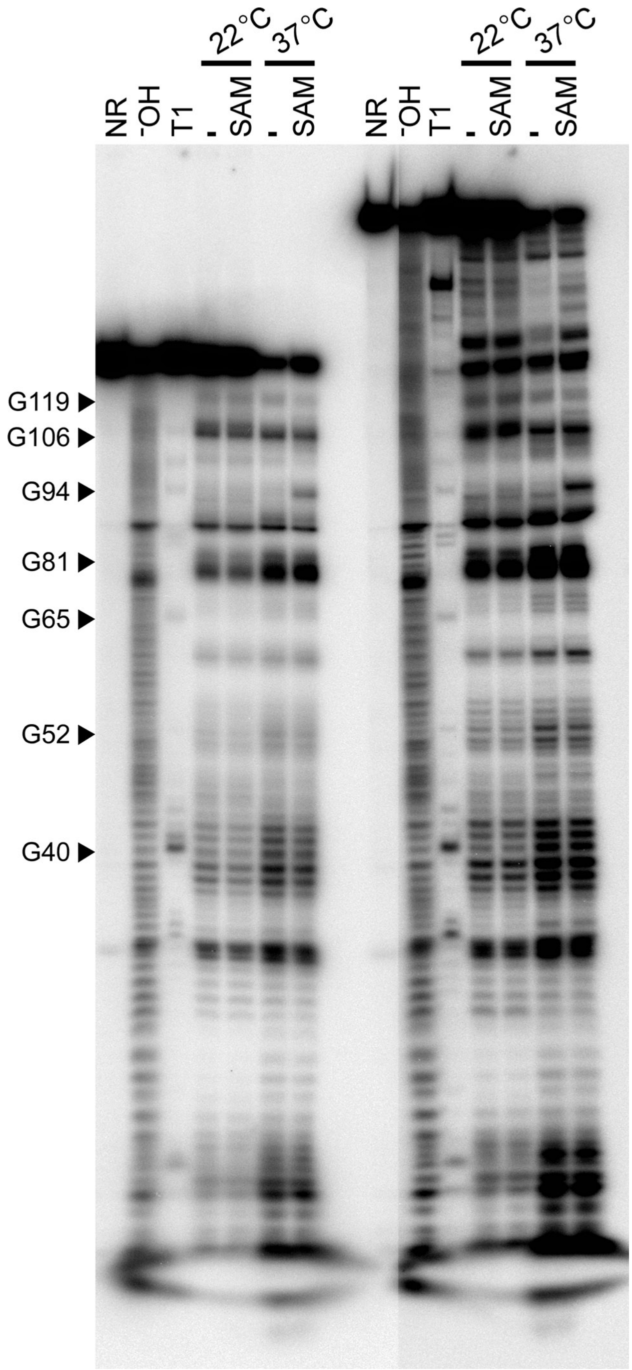

Supplementary Fig. 5 | In-line probing analysis of the aptamer domain and the full-length of SAM-I*Xcc* at room temperature (22 °C) and 37 °C. The same conventions apply as those shown in Fig. 1d. The in-line probing assays for the aptamer domain and the full-length were run on the left and right of the gel, respectively. Source data are provided as a Source Data file.

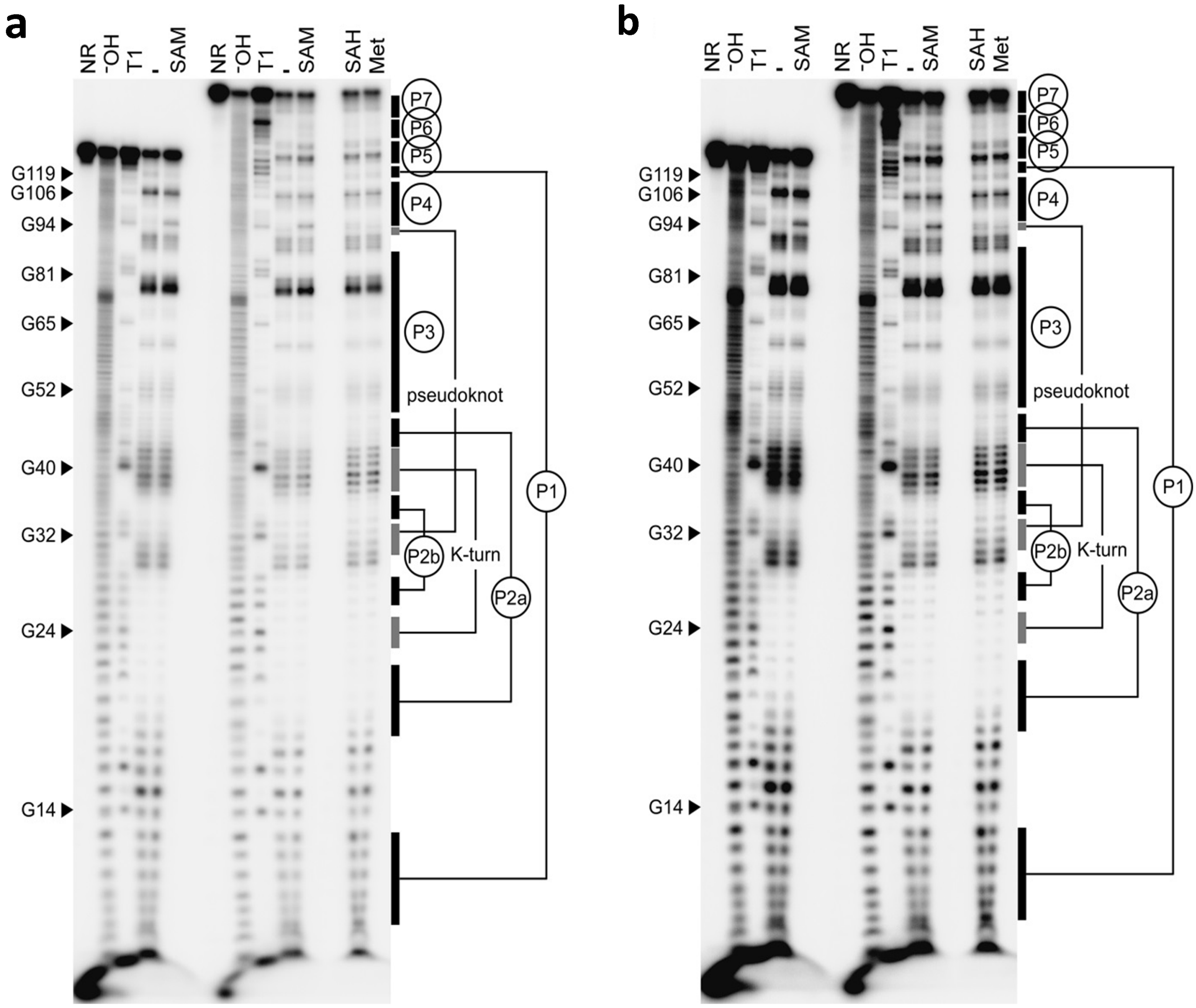

Supplementary Fig. 6 | In-line probing analysis of SAM-I$_{Xcc}$ 5′-region. The gels were run for a short time to examine the short 5′-cleaved signals, allowing analysis of the secondary structure of SAM-I$_{Xcc}$ at the 5′-end. **(a)** and **(b)** are the same gel adjusted by different contrast to better present the bands with different levels of ligand-responsive modulation. The same conventions apply as those shown in-line probing analysis seen in Fig. 1d. Source data are provided as a Source Data file.

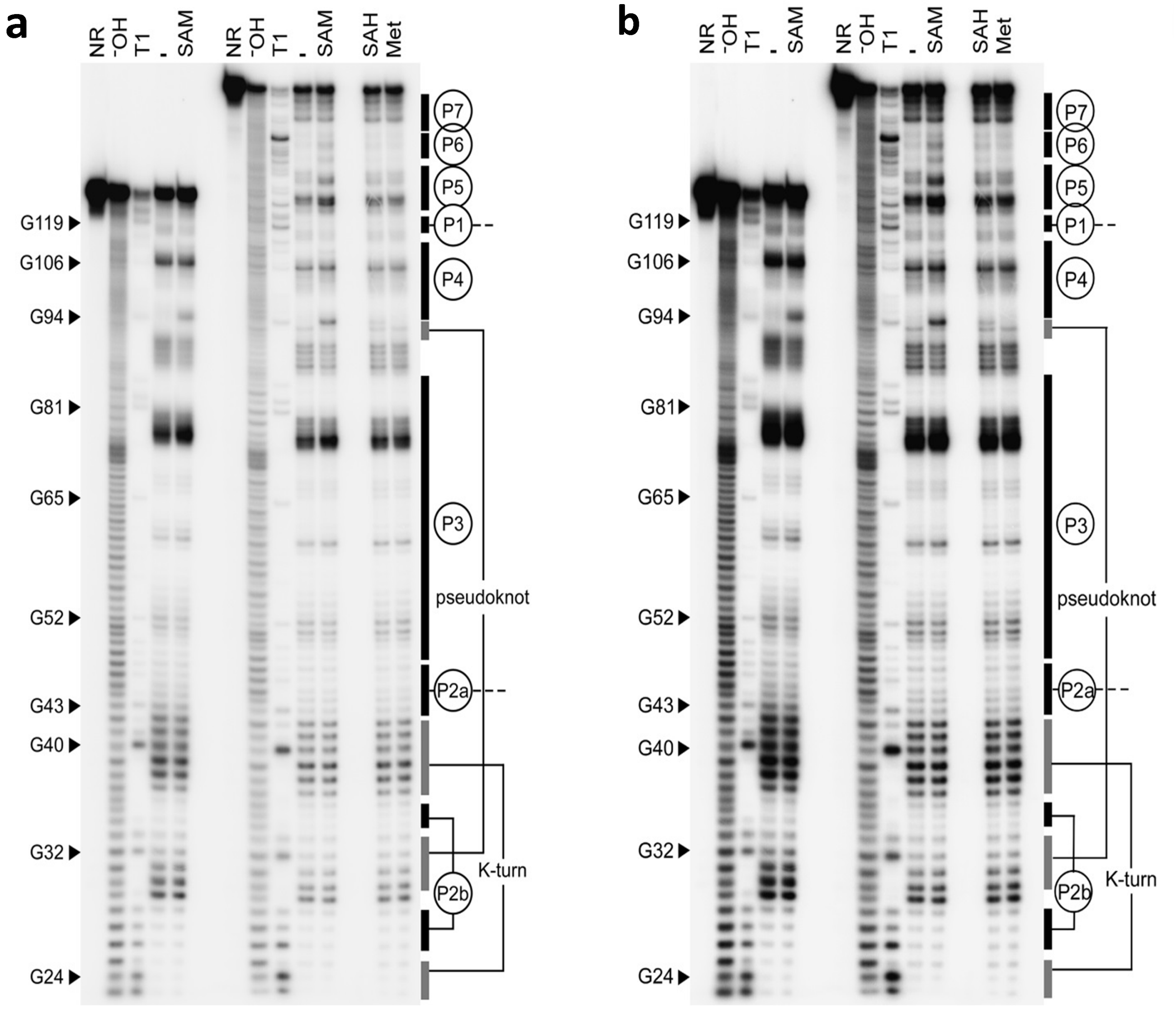

Supplementary Fig. 7 | In-line probing analysis of SAM-I*Xcc* middle region. The gels were run for longer time than those in Supplementary Fig. 6 to better separate the cleaved signals corresponding to the middle region of SAM-I*Xcc*, allowing analysis of the secondary structure there. **(a)** and **(b)** are the same gel adjusted by different contrast to better present the bands with different levels of ligand-responsive modulation. The same conventions apply as those shown in-line probing analysis seen in Fig. 1d. Note that **(b)** was also presented in Fig. 1d. Source data are provided as a Source Data file.

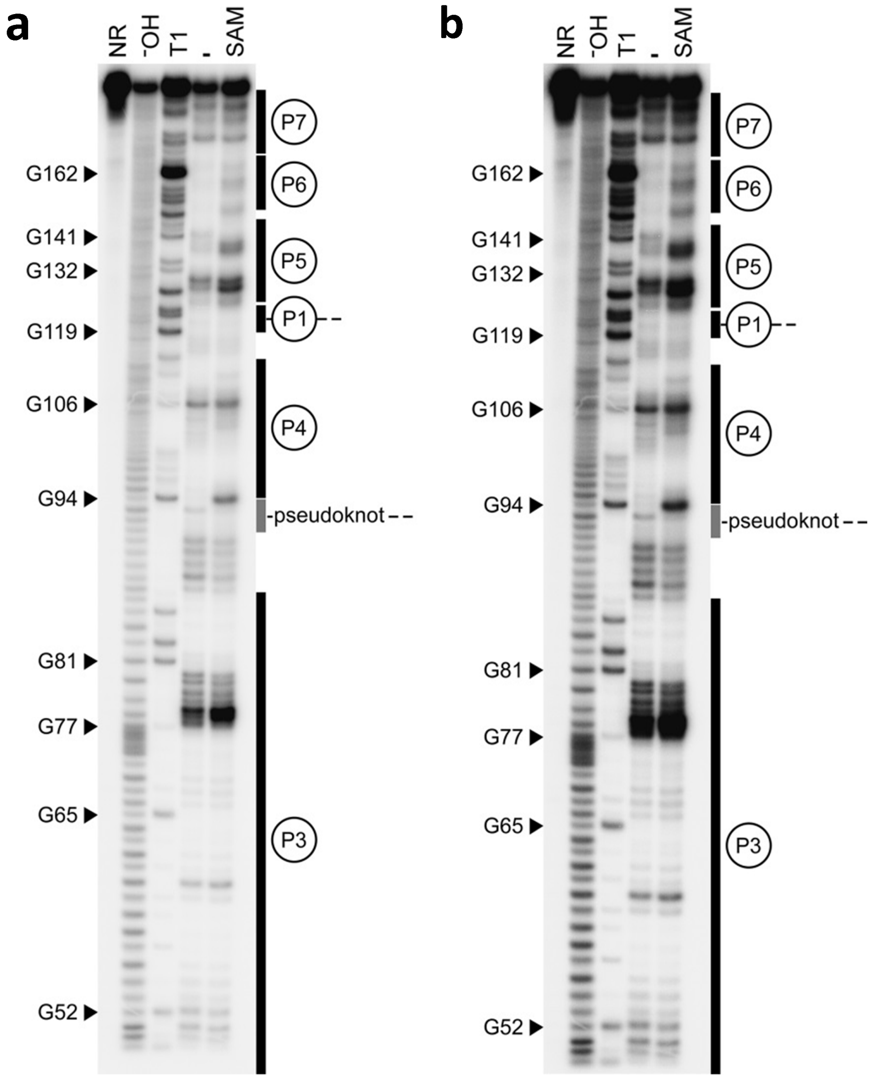

Supplementary Fig. 8 | In-line probing analysis of SAM-I*Xcc*3′-region. The gels were run for longer time than those shown in Supplementary Fig. 6 to better separate the cleaved signals at the 3′-end of SAM-I*Xcc*, allowing analysis of the secondary structure there. Only the full-length SAM-I*Xcc* was studied. **(a)** and **(b)** are the same gel adjusted by different contrast to better present the bands with different levels of ligand-responsive modulation. The same conventions apply as those shown in-line probing analysis seen in Fig. 1d. Source data are provided as a Source Data file.

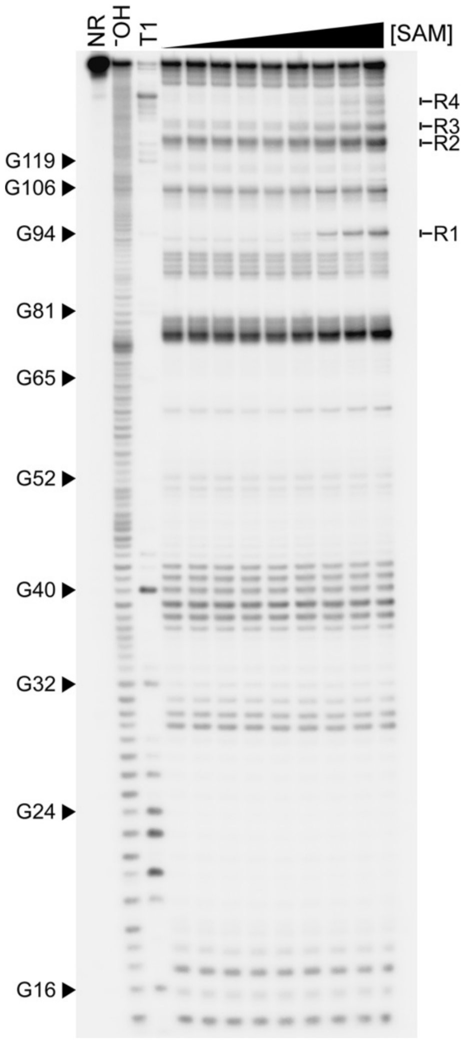

Supplementary Fig. 9 | In-line probing assay for the full-length SAM-I*Xcc* exposed to various concentrations of SAM. From left to right, the tested SAM concentrations are 0, 10 pM, 100 pM, 1 nM, 10 nM, 100 nM, 1 μM, 10 μM, 100 μM. R1-4 are the regions with apparent SAM modulation which were used to generate the K_D value in Fig. 1e. The same conventions apply as those shown in-line probing analysis seen in Fig. 1d. Source data are provided as a Source Data file.

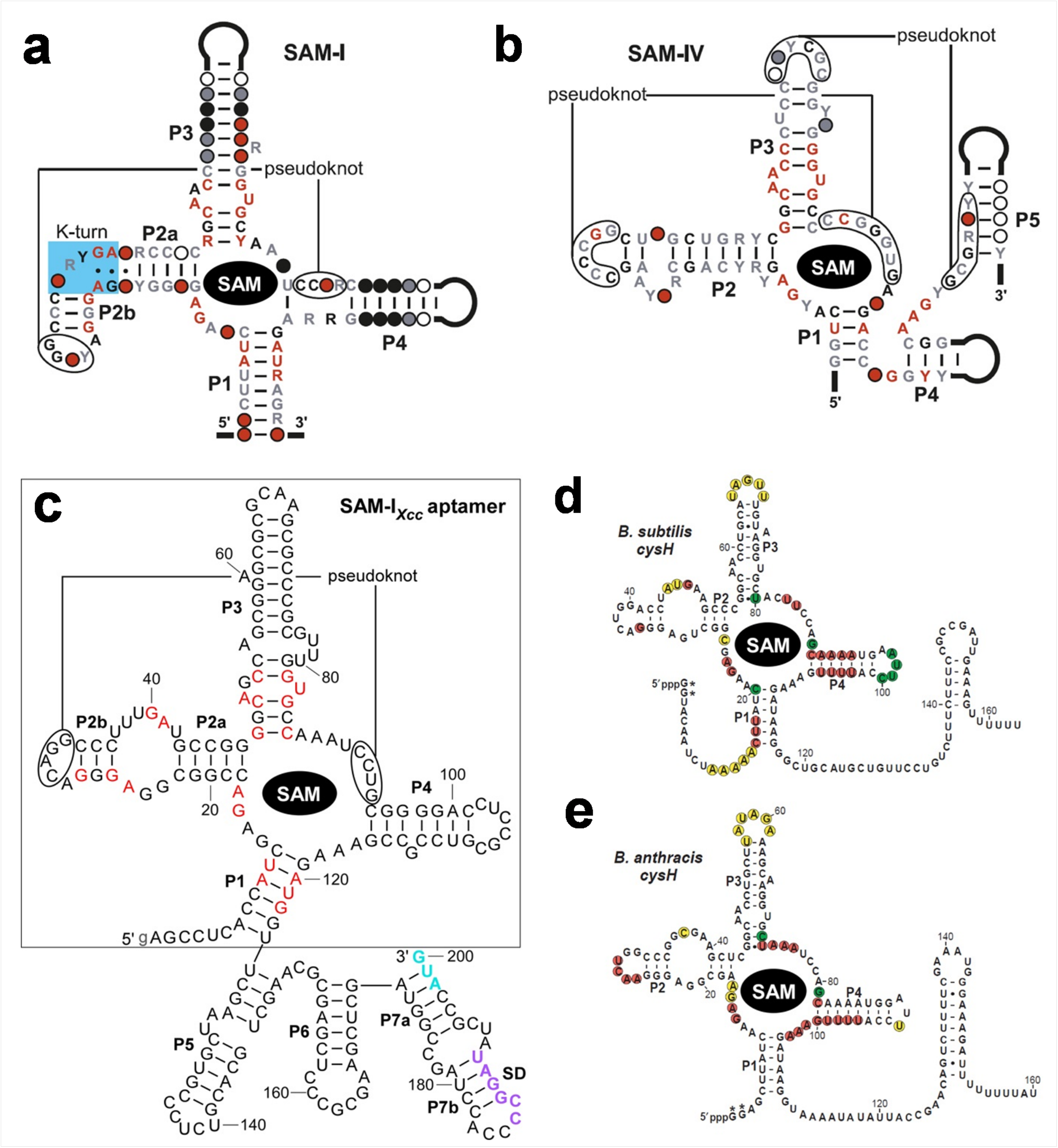

Supplementary Fig. 10 | Comparison of SAM-I$_{Xcc}$ with the reported SAM-I and SAM-IV riboswitches. (a)and **(b)**SAM-I and SAM-IV consensus models, taken from the review by Wang and Breaker[18] with a copyright permission issued by Marketplace™ (License ID: 1026781-3). **(c)** The sequence and secondary structure of SAM-I$_{Xcc}$ in the presence of SAM. Red nucleotides refer to the highly conserved ones in the SAM-I model shown in **(a)**. **(d)** and **(e)** The sequence and secondary structure of SAM-I riboswitch representatives in *B. subtilis* and *B. anthracis*, taken from the article

by Winkler and associates[14] with a copyright permission issued by RightsLink (License ID: 4802811191249).

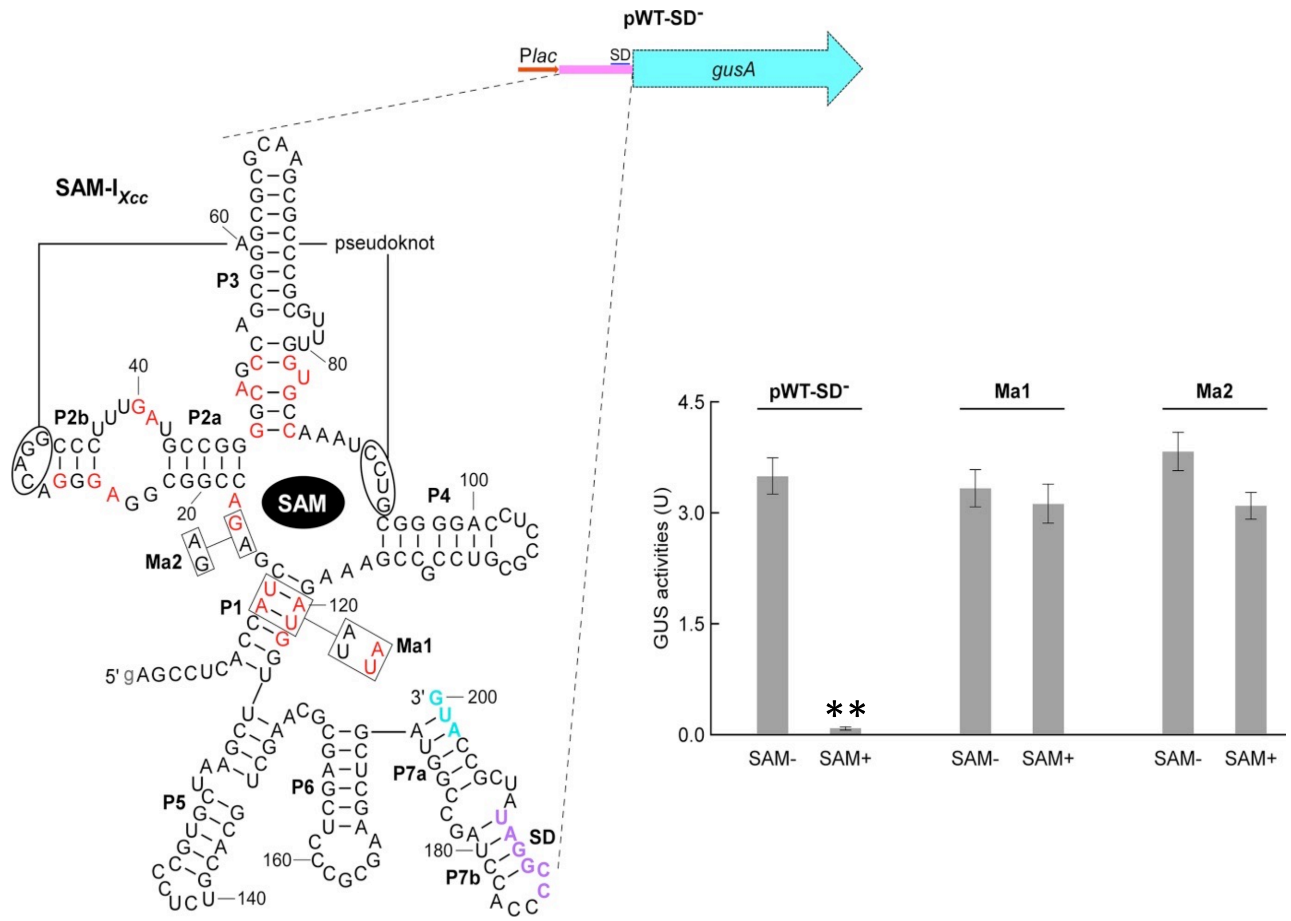

Supplementary Fig. 11 | Mutation of the potential SAM binding sites changing the SAM-I*Xcc* reporter from SAM responsive to unresponsive. The translational fusion reporter (pWT-SD$^-$, see Fig. 1b) was selected for this study. The left part shows the sequence and secondary structure of SAM-I*Xcc* riboswitch. Two mutations, Ma1 and Ma2, were designed to disrupt the reported binding sites of SAM-I riboswitches. The right part displays the GUS activities of the wild-type and the mutated reporters in response to SAM (300 μM). Data are presented as mean values ± SD from three biologically independent samples. Asterisks refer to the significant difference between SAM- and SAM+ of the same reporter strain, at $P< 0.01$ by Student's two-tailed *t*-test. Source data are provided as a Source Data file. The primer sets Plac-SAM-I-Ma1-F/SAM-I-R and Plac-SAM-I-Ma2-F/SAM-I-R (Supplementary Table 2) were used to construct the mutated reporters.

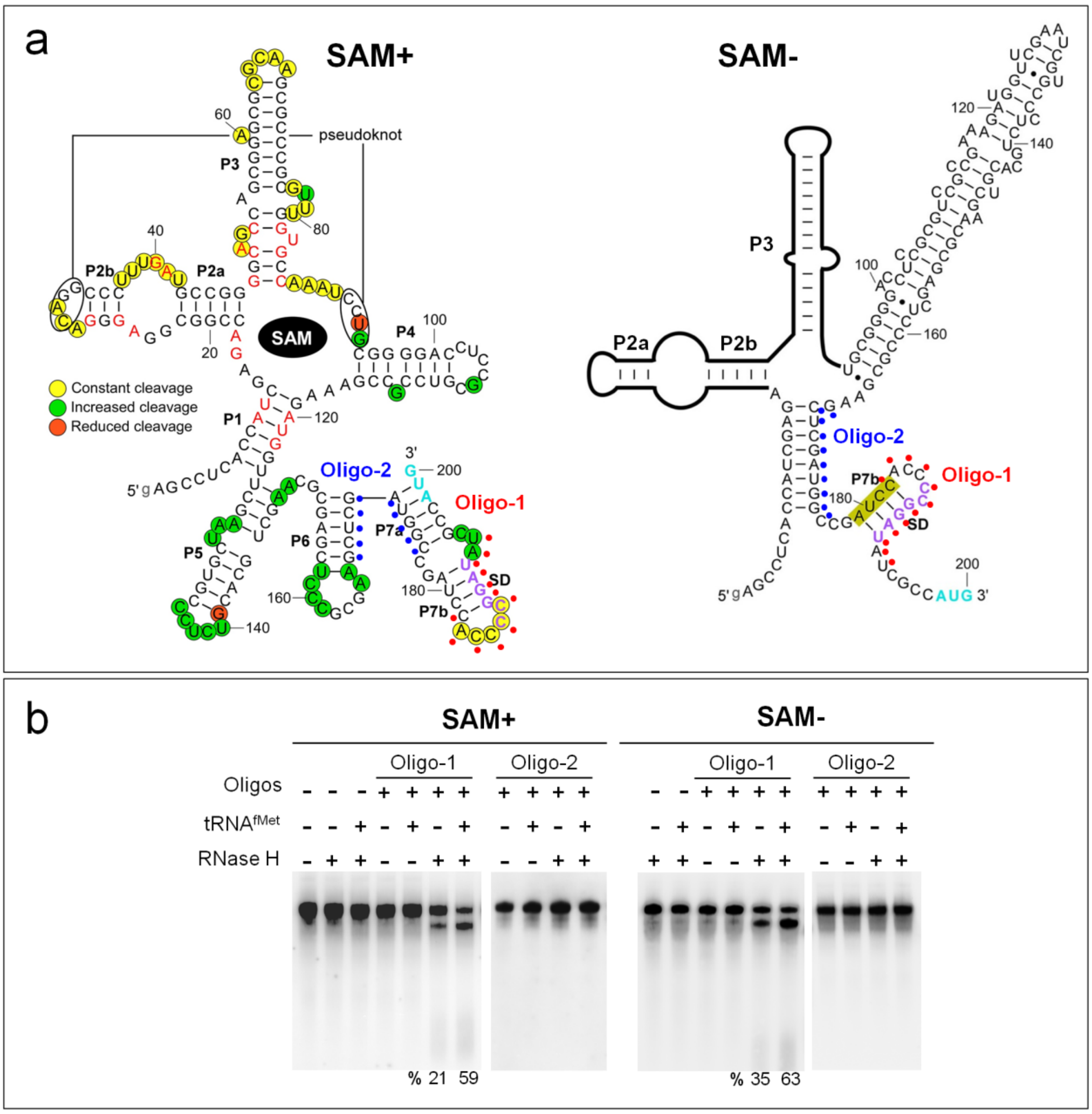

Supplementary Fig. 12 |Detection of conformational changes in SAM-I*Xcc* upon tRNA^{fMet} binding. (a) The secondary structure of SAM-I*Xcc* in the presence (SAM+, right) and absence (SAM-, left) of SAM. Nucleotides highlighted by red dots (Oligo-1: 5′-ATATCCGGGTG-3′) or blue dots (Oligo-2: 5′-GCCATCGAGC-3′) were chosen for the hybridization with a synthetic complementary DNA oligo. **(b)** Detection of conformational changes in the highlighted region upon tRNA^{fMet} binding by using the antisense DNA oligo and RNase H cleavage analysis. The percentage of the cleaved DIG-labeled SAM-I*Xcc* RNA was calculated using GelQuantNET software. Source data are provided as a Source Data file.

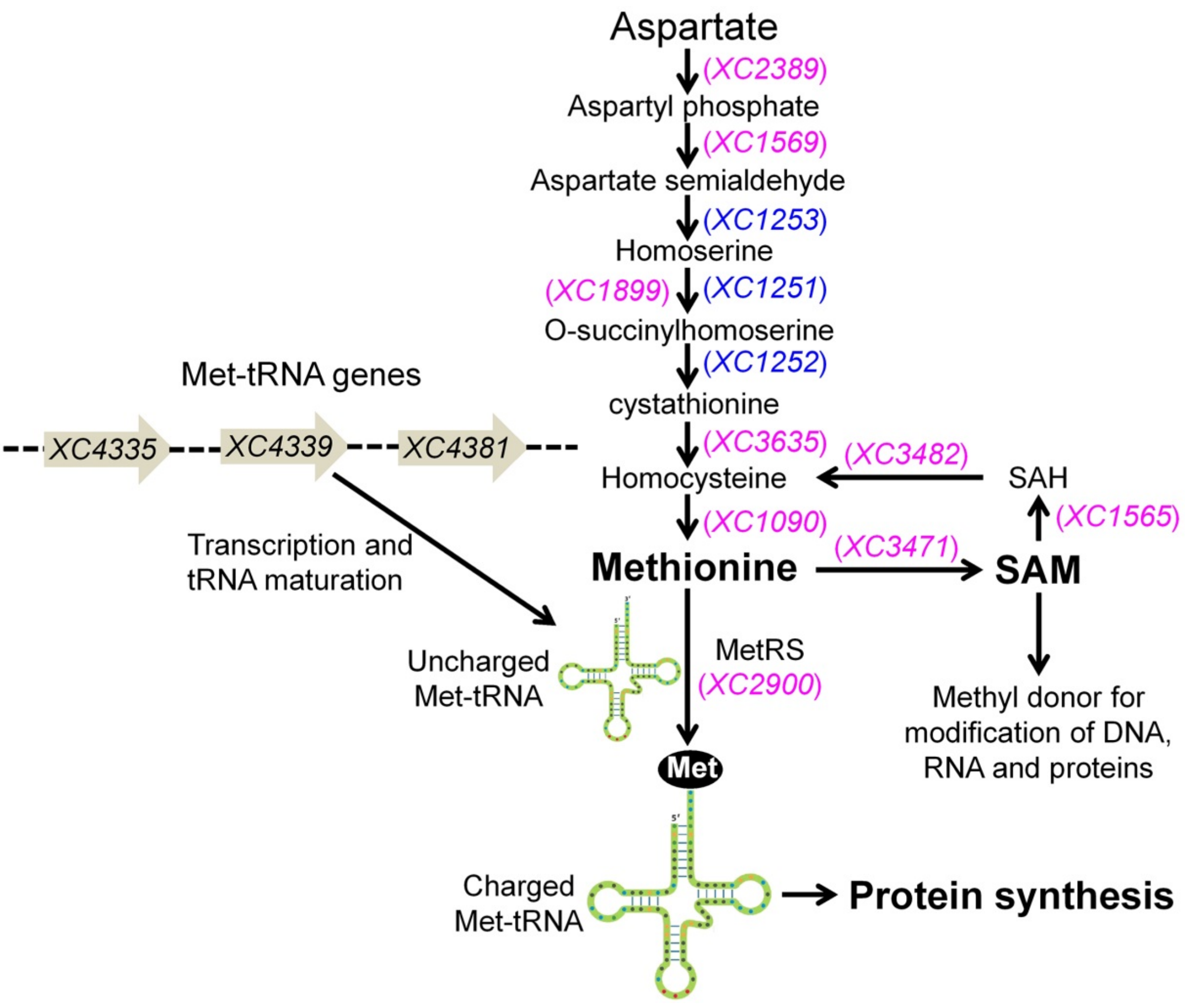

Supplementary Fig. 13 | A proposed pathway for the metabolism of methionine (Met), SAM and uncharged/charged Met-tRNA in *Xcc*. The pathway was established based on the known methionine biosynthesis pathways in bacteria[24] and the existence of related genes in the gnome of *Xcc* strain 8004[29]. SAM, S-adenosyl-methionine; SAH, S-adenosyl-homocysteine; MetRS, Methionyl-tRNA synthetase. *XCXXXX* refers to a gene of *Xcc* strain 8004 whose product catalyzes a reaction. Genes belonging to the *XC1251-XC1252-XC1253* operon are highlighted in blue and others are in purple.

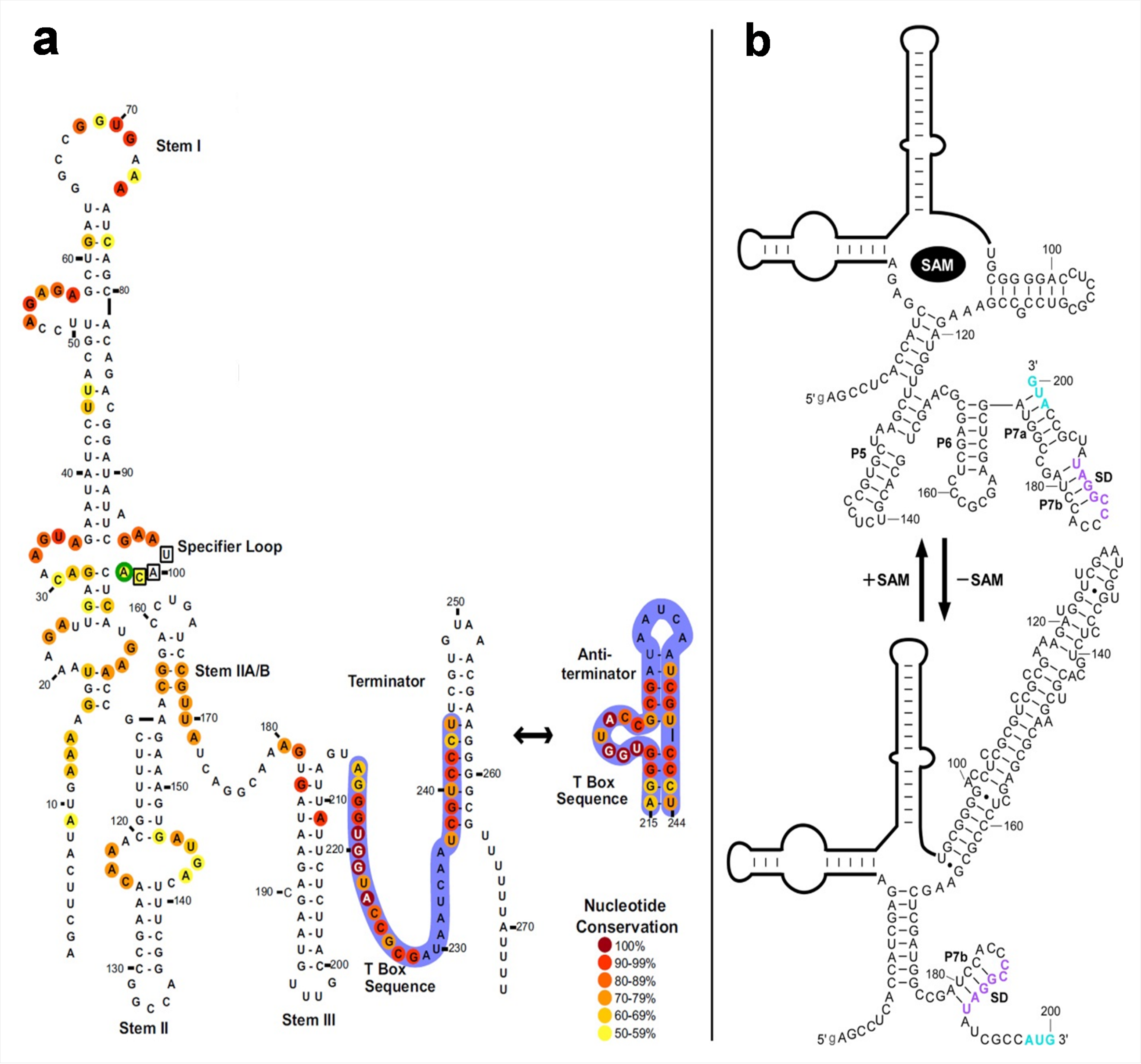

Supplementary Fig. 14 | Comparison of SAM-I*Xcc* with known T-box riboswitches. (a) The sequence and secondary structure model of a T-box riboswitch and its structural change upon sensing the corresponding tRNA, taken from a review by Gutiérrez-Preciadoand associates[8] with a copyright permission issued by RightsLink (License ID: 4802820123988). **(b)** The sequence and secondary structure model of SAM-I*Xcc* in SAM-bound and SAM-free states.

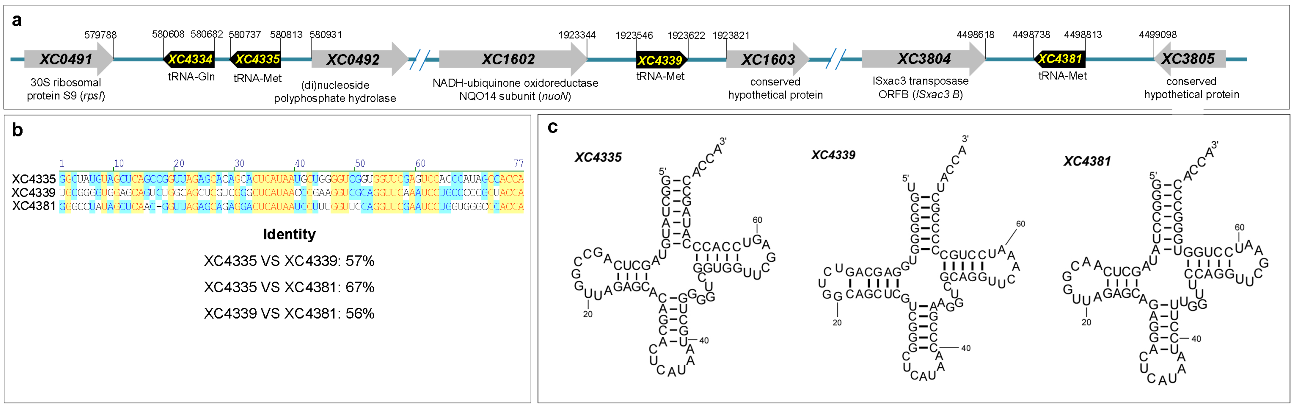

Supplementary Fig. 15 | Genome location, sequence and secondary structure of the three Met-tRNAs of *Xcc* strain 8004. (a) A schematic diagram showing the genomic location and genetic organization of $tRNA^{fMet}$ (*XC4339*), $tRNA^{Met1}$ (*XC4335*), and $tRNA^{Met2}$ (*XC4381*) genes in the genome of *Xcc* strain 8004. (**b**) Sequence comparison among the three Met-tRNAs of *Xcc* strain 8004. (**c**) The secondary structures of the three Met-tRNAs, which were predicted by using M-fold online software (http://unafold.rna.albany.edu/?q=mfold/RNA-Folding-Form).

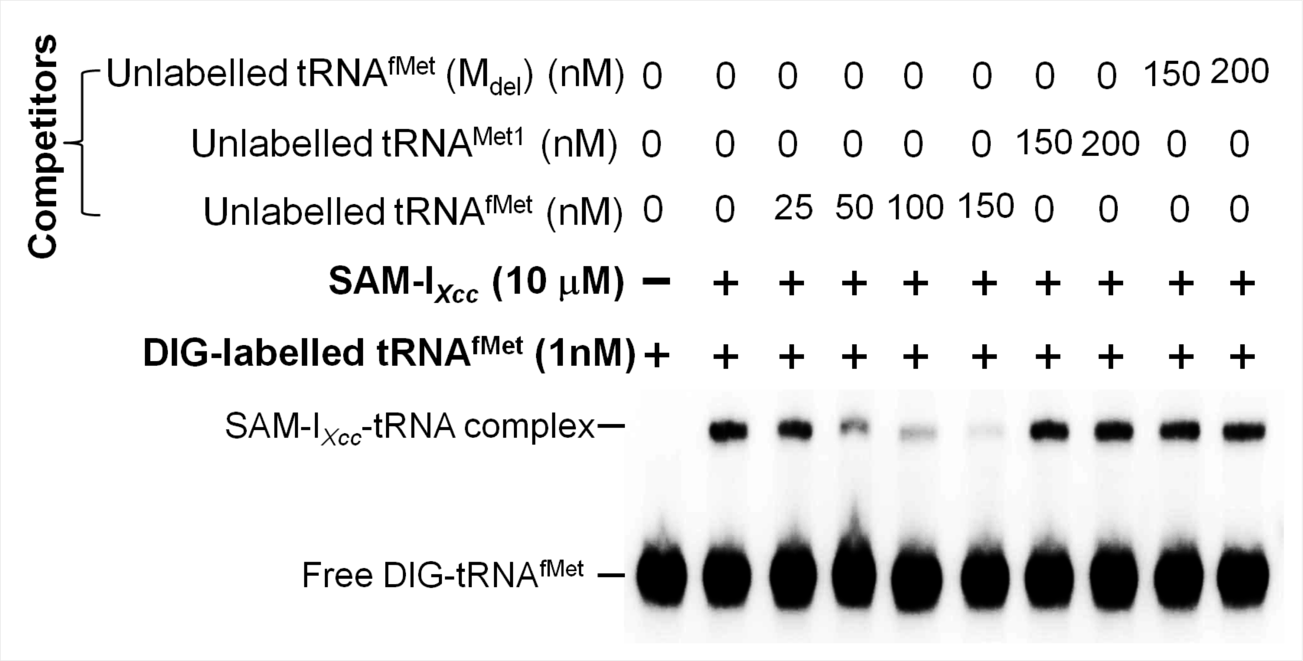

Supplementary Fig. 16 | Competition EMSA experiments with wild-type tRNA^{fMet}, mutant tRNA^{fMet} and tRNA^{Met1}. RNA molecule production and EMSA assay were performed as described in Methods. DIG-labeled tRNA^{fMet} (final concentration:1.0nM) was incubated without or with SAM-I$_{Xcc}$ (final concentration: 10 μM), in the presence of different amounts of unlabeled tRNA^{fMet} (25-200 fold excess, compared to the labeled tRNA^{fMet}), mutated tRNA^{fMet} [tRNA^{fMet}(Del)], and tRNA^{Met1}. Primers used for production of SAM-I$_{Xcc}$ and tRNAs were listed in Supplementary Table 2. Source data are provided as a Source Data file.

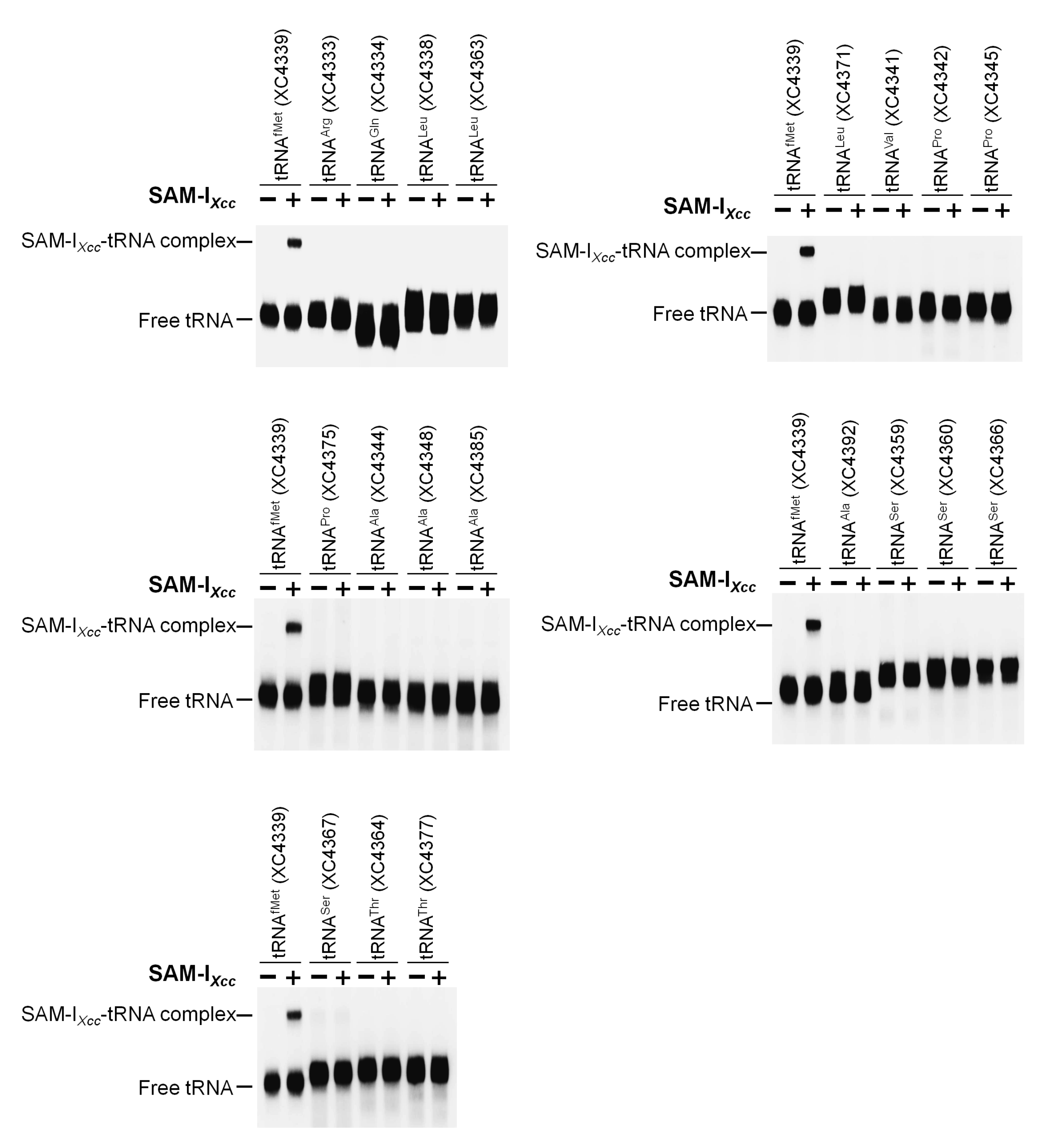

Supplementary Fig. 17 | EMSA detection showing that SAM-I$_{Xcc}$ does not bind with other tRNAs encoded in the genome of *Xcc* strain 8004. RNA molecule production and EMSA assay were performed as described in Methods. Primers used for production of SAM-I$_{Xcc}$ and tRNAs were listed in Supplementary Table 2. Detailed information of the tRNAs used in this experiment was listed in Supplementary Table 3. Source data are provided as a Source Data file.

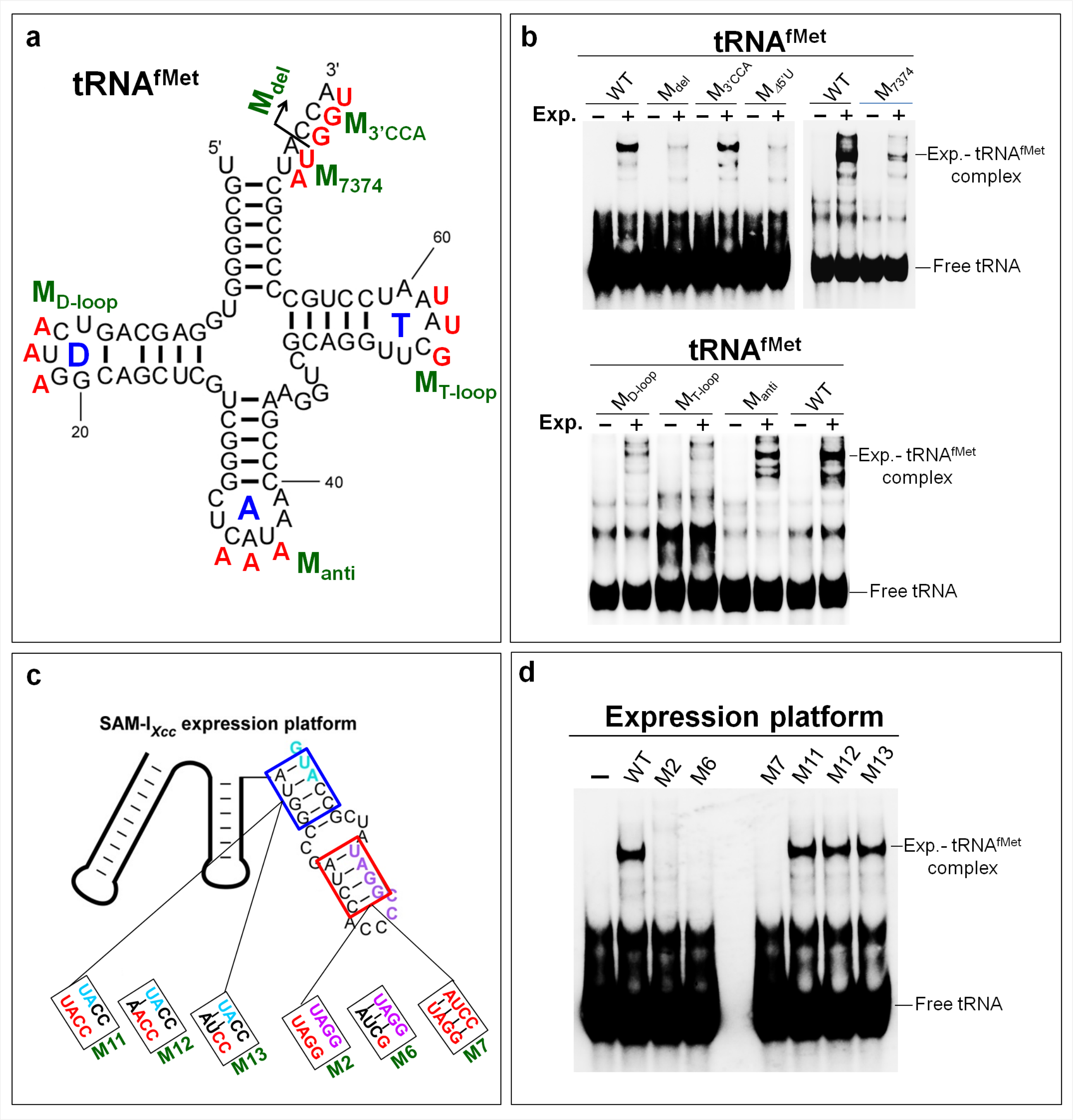

Supplementary Fig. 18 | Effect of mutations in tRNA^{fMet} or the expression platform of SAM-I*$_{Xcc}$* on the binding between SAM-I*$_{Xcc}$* and uncharged tRNA^{fMet} *in vitro*. (a) Overview of the mutations in tRMA^{fMet}. **(b)** EMSA detection of *in vitro* binding of the wild type (WT) and the mutant tRMA^{fMet} with the expression platform of SAM-I*$_{Xcc}$*. **(c)** Overview of the mutations in the expression platform of SAM-I*$_{Xcc}$*. **(d)** EMSA detection of *in vitro* binding of the WT and the mutant expression platforms with tRNA^{fMet}. EMSA assay was performed as described in Methods. The

primers used to construct the mutated $tRMA^{fMet}$ and the expression platform of SAM-I_{Xcc} are listed in Supplementary Table 2. Source data are provided as a Source Data file.

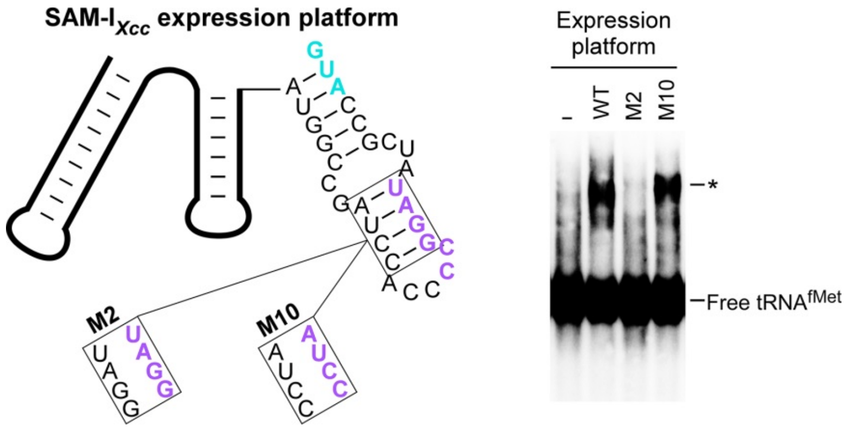

Supplementary Fig. 19| Negligible effect of the SD's GGAU→CCUA mutation on the binding of SAM-I*Xcc* with uncharged tRNA^{fMet} *in vitro*. Left: overview of the M2 and M10 mutations in the expression platform of SAM-I*Xcc*. Right: EMSA detection of *in vitro* binding of the wild type (WT) and the mutant expression platforms with tRNA^{fMet}. RNA molecule production and EMSA assay were performed as described in Methods. Primers used for production of the WT, the M2 mutant, and the M10 mutant expression platform were listed in Supplementary Table 2. *, complex of expression platform and tRNA^{fMet}. Source data are provided as a Source Data file.

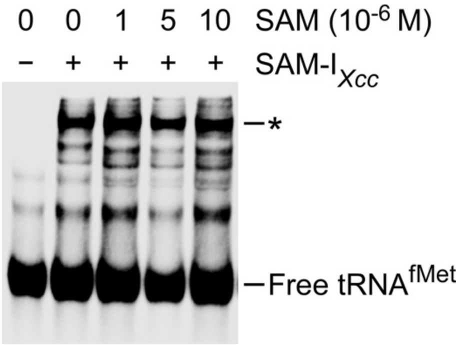

Supplementary Fig. 20| Negligible effect of SAM on the binding of SAM-I*Xcc* with uncharged tRNA^{fMet} *in vitro*. Different levels of SAM were added to the mixture of SAM-I*Xcc* and tRNA^{fMet}. EMSA assay was performed as described in Methods. *, complex of SAM-I*Xcc* and tRNA^{fMet}. Source data are provided as a Source Data file.

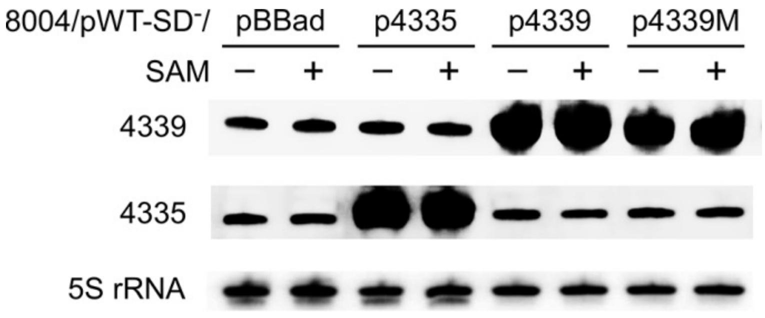

Supplementary Fig. 21| Confirmation of tRNA over-expression in the desired strains by Northern blotting. Reporter strains *Xcc* 8004/pWT-SD$^-$ carrying the series of plasmids (pBBad, p4335, p4339, p4339M) in Fig. 4c were grown in the minimal medium MMX (SAM-) or MMX supplemented with SAM (SAM+) to a final concentration of 2.5 μM. The expression level of tRNA^{fMet} (*XC4339*) and tRNA^{Met1} (*XC4335*) was examined by Northern blotting as described in Methods. 5S rRNA was used as a control. Source data are provided as a Source Data file.

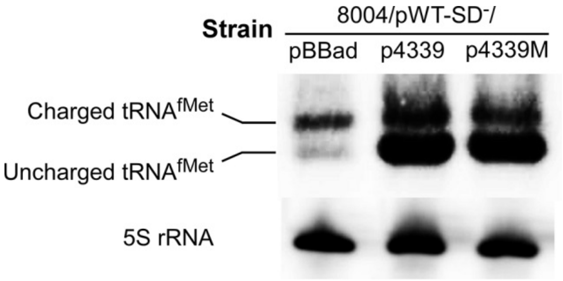

Supplementary Fig. 22| Confirmation of uncharged tRNA^{fMet} over-expression in the desired strains by Northern blotting. The level of charged and uncharged tRNA^{fMet} in the reporter strains *Xcc* 8004/pWT-SD^{-} carrying the vector pBBad or a recombinant pBBad expressing tRNA^{fMet} (p4339, p4339M) was estimated by Northern blotting as described in Methods. 5S rRNA was used as a control. Source data are provided as a Source Data file.

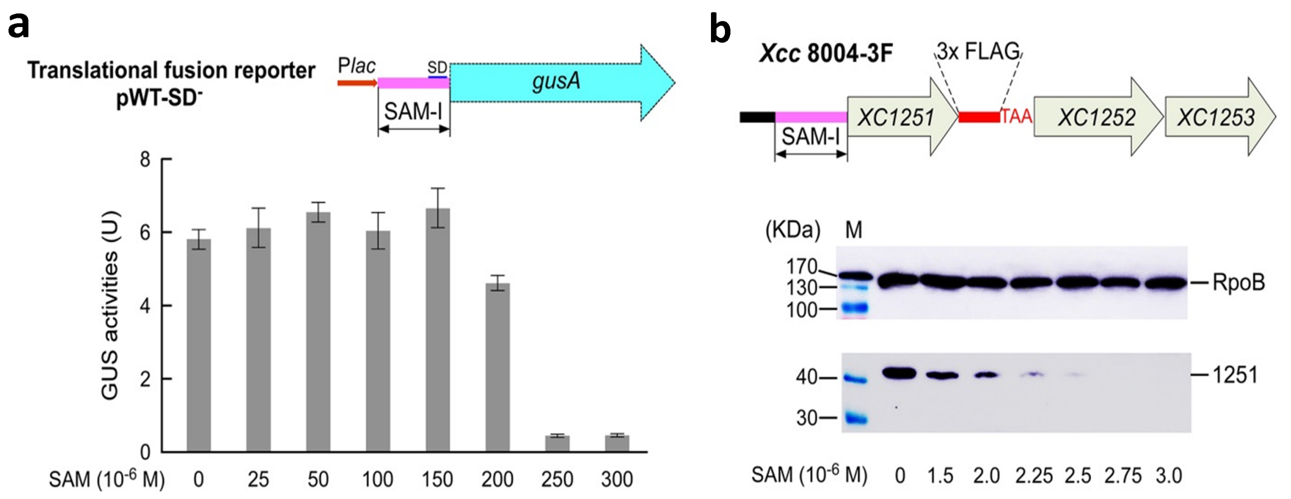

Supplementary Fig. 23| Determination of the minimal inhibitory concentration of SAM for SAM-I$_{Xcc}$. (a) Effect of SAM concentration on the GUS activity of the GUS reporter strain *Xcc* 8004/pWT-SD$^-$. The strain was cultured in the minimal medium MMX (SAM-) or MMX supplemented with SAM to a final concentration from 25 to 300 μM. GUS activities were tested 30 hours post-inoculation. Data are presented as mean values ± SD from three biologically independent samples. GUS assay is detailed in the Methods. **(b)** Effect of SAM concentration on XC1251-3F protein expression in strain *Xcc* 8004-3F. The strain was cultured in MMX (SAM-) or MMX supplemented with SAM to a final concentration from 1.5 to 3.0 μM. Cells were collected 30 hours post-inoculation and total proteins were isolated from the cells for Western blotting analysis. RNA polymerase β sub-unit (RpoB) protein was used as a control. Source data are provided as a Source Data file.

The SAM concentrations that completely inhibit GUS activity of the GUS reporter strain (8004/pWT-SD$^-$) and the expression of XC1251-3F protein in the FLAG-tagging strain (8004-3F) are ~250 μM and ~2.5 μM, respectively. The difference in SAM inhibitory concentrations indicates that the *met* operon mRNA is involved in the SAM-responsive regulation. It is likely that the inhibition of the translation initiation of *met* operon mRNA triggers the mRNA degradation by the RNase that

specifically recognizes them. However, it is also possible that the difference is simply due to the copy number of SAM-I_{Xcc} (8004-3F strain contains only a single copy while 8004/pWT-SD^- strain contains multiple copy of SAM-I_{Xcc}).

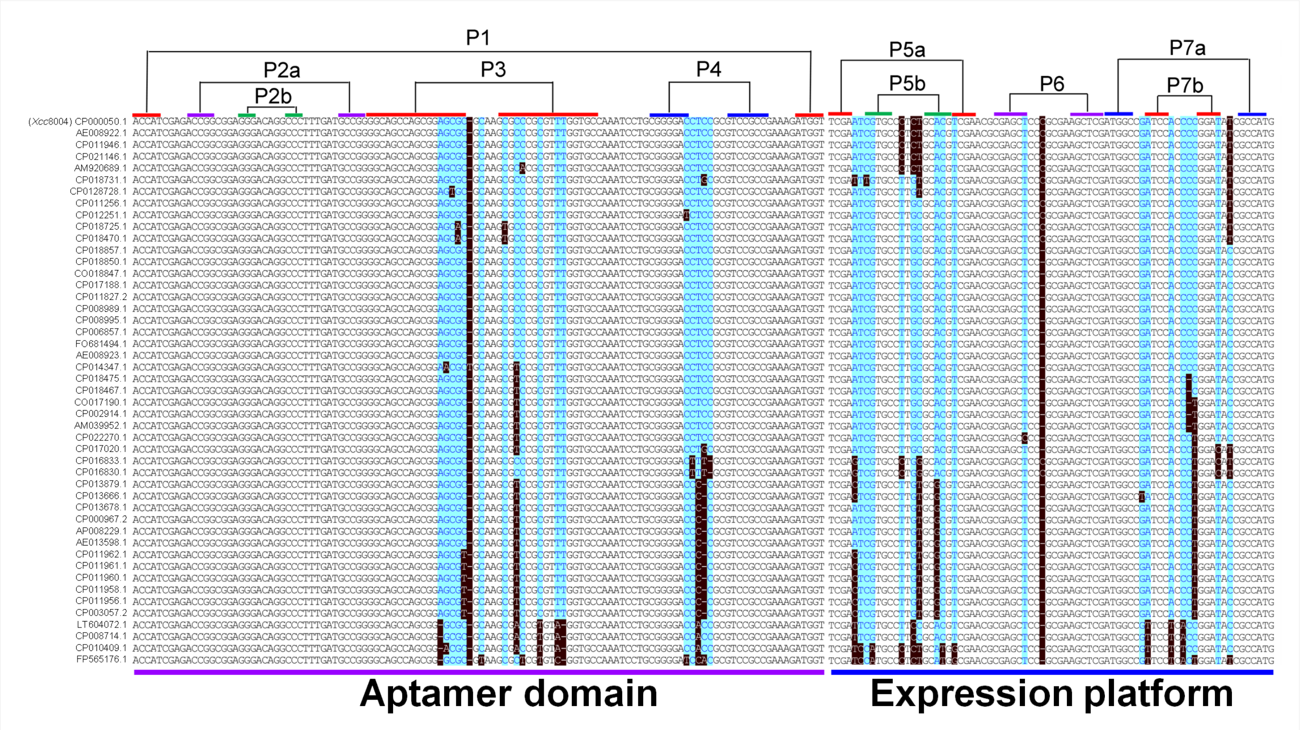

Supplementary Fig. 24 | Alignment of SAM-I$_{Xcc}$ coding sequence and the putative 5′UTR of *metA* (homoserine O-acetyltransferase) gene from different *Xanthomonas* species and strains. All sequences were from the sequence databases on the National Center for Biotechnology Information website. Blue boxes indicate the region with nucleotide variations and black boxes denote the nucleotide that is different from SAM-I$_{Xcc}$. The base pairing regions (P1 to P7) are indicated at the top. The right side shows genome GenBank accession number, and the corresponding species and strains were listed in Supplementary Table 4.

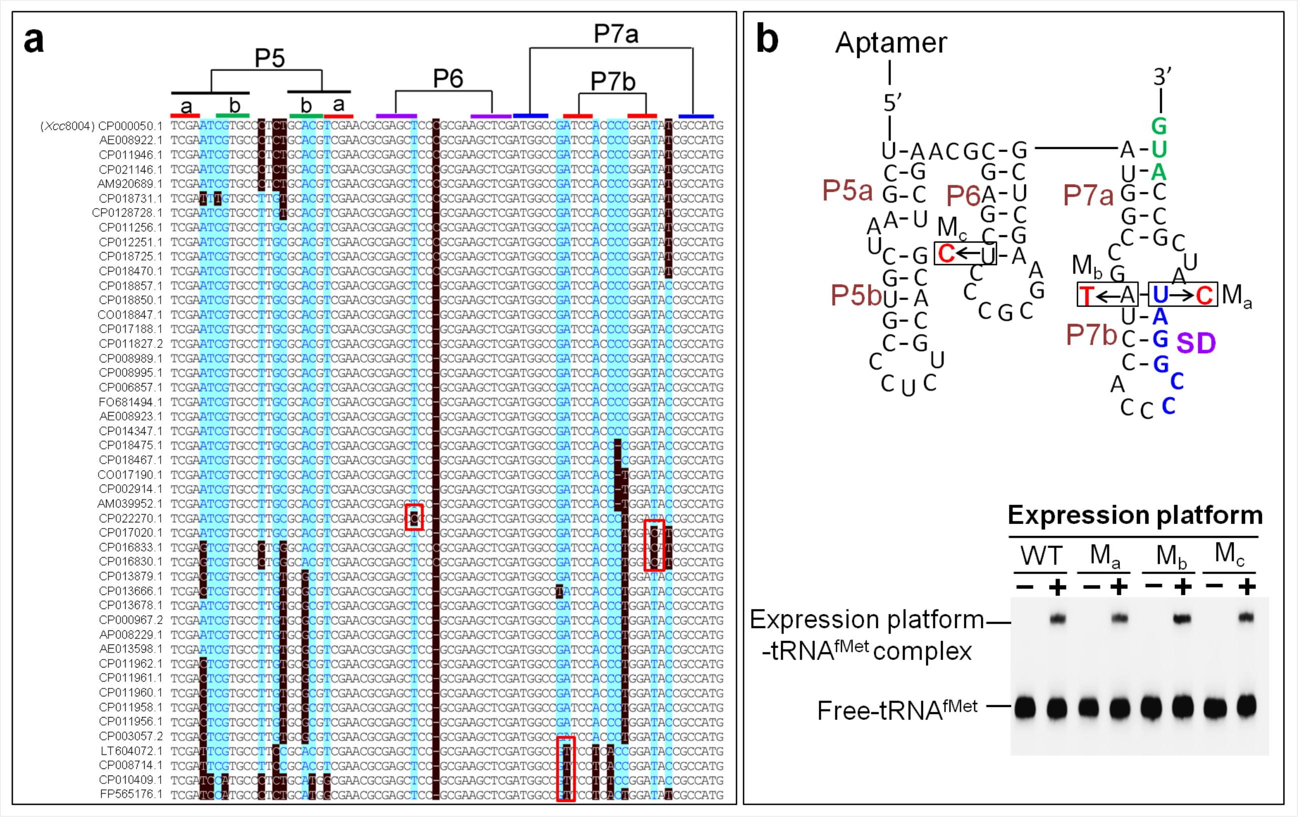

Supplementary Fig. 25 | EMSA detection showing that a single nucleotide mutation in P6 and P7 regions does not affect the binding of SAM-I$_{Xcc}$ with tRNAf^{Met}. (a) Alignment of the coding sequence of the expression platform of SAM-I$_{Xcc}$ homologues from different *Xanthomonas* species and strains. The red boxed indicates unconserved nucleotides in P6 and P7 stems. **(b)** EMSA detection of the binding of tRNA^{fMet} with the wild-type expression platform and the expression platform with a mutation in the nucleotide corresponding to red boxed nucleotide indicated in panel a. RNA preparation and EMSA assay were performed as described in Methods. Primers used for RNA production in this experiment are listed in Supplementary Table 2. Source data are provided as a Source Data file.

Supplementary Table 1 | Bacterial strains and plasmids used in this work.

Strains and plasmids	Relevant characteristics*	Ref.
***E.coli* strains**		
JM109	*RecA*1, *endA*1, *gyrA*96, *thi*, *supE*44, *relA*1△ (*lac-proAB*)/F' [*traD*36, *lacI*^{q}, *lacZ*△M15]	49
***Xcc* strains**		
8004	Wild type, Rif^r	44
**1201PK2**	As 8004 but *XC1251* (*metA*)::pK18mob, Rif^r, Kan^r	26
8004/pWT-SD$^+$	8004 containing the plasmid pWT-SD$^+$, Rif^r, Tc^r	26
8004/pWT-SD$^-$	8004 containing the plasmid pWT-SD$^-$, Rif^r, Tc^r	26
8004/pM1-SD$^-$	8004 containing the plasmid pM1-SD$^-$, Rif^r, Tc^r	This work
8004/pM2-SD$^-$	8004 containing the plasmid pM2-SD$^-$, Rif^r, Tc^r	This work
8004/pM(1+2)-SD$^-$	8004 containing the plasmid pM(1+2)-SD$^-$, Rif^r, Tc^r	This work
8004/p0-SD+	8004 containing the plasmid p0-SD$^+$, Rif^r, Tc^r	This work
8004/pTrp-SD+	8004 containing the plasmid pTrp-SD$^+$, Rif^r, Tc^r	This work
8004/p5-SD+	8004 containing the plasmid p5-SD$^+$, Rif^r, Tc^r	This work
8004/p6-SD+	8004 containing the plasmid p6-SD$^+$, Rif^r, Tc^r	This work
8004/p7-SD+	8004 containing the plasmid p7-SD$^+$, Rif^r, Tc^r	This work
8004/p56-SD+	8004 containing the plasmid p56-SD$^+$, Rif^r, Tc^r	This work
8004/p67-SD+	8004 containing the plasmid p67-SD$^+$, Rif^r, Tc^r	This work
8004/p567-SD+	8004 containing the plasmid p567-SD$^+$, Rif^r, Tc^r	This work
8004-3F	As 8004, but a 3xFlag coding sequence was inserted in to the XC1251 gene just before the stop codon TAA, Rif^r	26
8004-3F/p4335	XC1251-3F containing the plasmid p4335, Rif^r, Kan^r	This work
8004-3F/p4339	XC1251-3F containing the plasmid p4339, Rif^r, Kan^r	This work
8004-3F/p4339M	XC1251-3F containing the plasmid p4339M, Rif^r, Kan^r	This work
8004/pWT-SD$^-$/p4335	8004/pWT-SD$^-$containing the plasmid p4335, Rif^r, Tc^r, Kan^r	This work
8004/pWT-SD$^-$/p4339	8004/pWT-SD$^-$containing the plasmid p4339, Rif^r, Tc^r, Kan^r	This work
8004/pWT-SD$^-$/p4339M	8004/pWT-SD$^-$containing the plasmid p4339M, Rif^r, Tc^r, Kan^r	This work
**Plasmids**		
pLAFR6	Broad host range cloning vector, Tc^r	50
pWT-SD$^+$	pLAFR6 contains the WT SAM-I$_{Xcc}$-SD$^+$*gusA* fusion fragment, Tc^r	26
pWT-SD$^-$	pLAFR6 contains the WT SAM-I$_{Xcc}$-SD$^-$*gusA* fusion fragment, Tc^r	26
pM1-SD$^-$	pLAFR6 contains the M1 SAM-I$_{Xcc}$-SD$^-$*gusA* fusion fragment, Tc^r	This work
pM2-SD$^-$	pLAFR6 contains the M2 SAM-I$_{Xcc}$-SD$^-$*gusA* fusion fragment, Tc^r	This work
pM(1+2)-SD$^-$	pLAFR6 contains the M1+2 SAM-I$_{Xcc}$-SD$^-$*gusA* fusion fragment, Tc^r	This work

p0-SD+	pLAFR6 contains the *lac* promoter-SD$^+$*gusA* fusion fragment, Tc^r	This work
pTrp-SD+	pLAFR6 contains the *lac* promoter-trp terminator-SD$^+$*gusA* fusion fragment, Tc^r	This work
p5-SD+	pLAFR6 contains the *lac* promoter-hairpin P5-SD$^+$*gusA* fusion fragment, Tc^r	This work
p6-SD+	pLAFR6 contains the *lac* promoter-hairpin P6-SD$^+$*gusA* fusion fragment, Tc^r	This work
p7-SD+	pLAFR6 contains the *lac* promoter-hairpin P7-SD$^+$*gusA* fusion fragment, Tc^r	This work
p56-SD+	pLAFR6 contains the *lac* promoter-hairpin P5+P6-SD$^+$*gusA* fusion fragment, Tc^r	This work
p67-SD+	pLAFR6 contains the *lac* promoter-hairpin P6+P7-SD$^+$*gusA* fusion fragment, Tc^r	This work
p567-SD+	pLAFR6 contains the *lac* promoter-hairpin P5+P6+P7-SD$^+$*gusA* fusion fragment, Tc^r	This work
pBBad	Over-expression vector, Kan^r	51
p4335	pBBad contains *XC4335* coding sequence, Kan^r	This work
p4339	pBBad contains *XC4339* coding sequence, Kan^r	This work
p4339M	pBBad contains the *XC4339* A73U74 mutation, Kan^r	This work

*Rif^r, Kan^r, and Tc^r refer to rifampicin-, kanamycin-, and tetracycline-resistance, respectively.

Supplementary Table 2 | Primer sets and DNA oligos used in this work.

Primer pairs/oligos	Sequence (5′ to 3′)*	Purpose
AE-T7-F/AE-R	TAATACGACTCACTATAGAGCCTCACCATCGAGACCGGCG/GAGGGCATGGCGATATCCGGGGTGG	For generating full-length SAM-I$_{Xcc}$ RNA
AE-T7-F/A-R	TAATACGACTCACTATAGAGCCTCACCATCGAGACCGGCG/ACCATCTTTCGGCGGACGCGG	For generating the aptamer of SAM-I$_{Xcc}$ RNA
E-T7-F/AE-R	TAATACGACTCACTATAGTCGAATCGTGCCCTCTGCAC/GAGGGCATGGCGATATCCGGGGTGG	For generating the expression platform of SAM-I$_{Xcc}$ RNA
E-T7-F/E-M2-R	TAATACGACTCACTATAGTCGAATCGTGCCCTCTGCAC/CATGGCGATATCCGGGGTCCTACGGCCATCGAGCTTCGCGGG	For generating the expression platform with M2 mutation
E-T7-F/E-M3-R	TAATACGACTCACTATAGTCGAATCGTGCCCTCTGCAC/CATGGCGATATCCGGGGTGGTACGGCCATCGAGCTTCGCGGG	For generating the expression platform with M3 mutation
E-T7-F/E-M4-R	TAATACGACTCACTATAGTCGAATCGTGCCCTCTGCAC/CATGGCGATATCCGGGGTCCATCGGCCATCGAGCTTCGCGGG	For generating the expression platform with M4 mutation
E-T7-F/E-M5-R	TAATACGACTCACTATAGTCGAATCGTGCCCTCTGCAC/CATGGCGATATCCGGGGTGCATCGGCCATCGAGCTTCGCGGG	For generating the expression platform with M5 mutation
E-T7-F/E-M6-R	TAATACGACTCACTATAGTCGAATCGTGCCCTCTGCAC/CATGGCGATATCCGGGGTCGATCGGCCATCGAGCTTCGCGGG	For generating the expression platform with M6 mutation
E-T7-F/E-M7-R	TAATACGACTCACTATAGTCGAATCGTGCCCTCTGCAC/CATGGCGATTAGGGGGGTCCTACGGCCATCGAGCTTCGCGGG	For generating the expression platform with M7 mutation
E-T7-F/E-M8-R	TAATACGACTCACTATAGTCGAATCGTGCCCTCTGCAC/CATGGCGATATGGGGGGTCCATCGGCCATCGAGCTTCGCGGG	For generating the expression platform with M8 mutation
E-T7-F/E-M9-R	TAATACGACTCACTATAGTCGAATCGTGCCCTCTGCAC/CATGGCGATATGCGGGGTCGATCGGCCATCGAGCTTCGCGGG	For generating the expression platform with M9 mutation
E-T7-F/AE-R	TAATACGACTCACTATAGTCGAATCGTGCCCTCTGCAC/GAGGGCATGGCGATATCCGGGGTGG	For generating the expression platform of SAM-I$_{Xcc}$ RNA
E-T7-F/E-M2-R	TAATACGACTCACTATAGTCGAATCGTGCCCTCTGCAC/CATGGCGATATCCGGGGTCCTACGGCCATCGAGCTTCGCGGG	For generating the expression platform with M2 mutation
E-T7-F/E-M6-R	TAATACGACTCACTATAGTCGAATCGTGCCCTCTGCAC/CATGGCGATATCCGGGGTCGATCGGCCATCGAGCTTCGCGGG	For generating the expression platform with M6 mutation
E-T7-F/E-M7-R	TAATACGACTCACTATAGTCGAATCGTGCCCTCTGCAC/CATGGCGATTAGGGGGGTCCTACGGCCATCGAGCTTCGCGGG	For generating the expression platform with M7 mutation

E-T7-F/E-M11-R	TAATACGACTCACTATAGTCGAATCGTGCCCTCTGCAC/CATGGCGATATCCGGGGTGGATCGGGGTACGAGCTTCGCGGG	For generating the expression platform with M11 mutation
E-T7-F/E-M12-R	TAATACGACTCACTATAGTCGAATCGTGCCCTCTGCAC/CATGGCGATATCCGGGGTGGATCGGGGTTCGAGCTTCGCGGG	For generating the expression platform with M12 mutation
E-T7-F/E-M13-R	TAATACGACTCACTATAGTCGAATCGTGCCCTCTGCAC/CATGGCGATATCCGGGGTGGATCGGGGATCGAGCTTCGCGGG	For generating the expression platform with M13 mutation
E-T7-F/EMa-R	TAATACGACTCACTATAGTCGAATCGTGCCCTCTGCAC/CATGGCGATGTCCGGGGTGG	For generating the M_a mutant expression platform RNA
E-T7-F/EM$_b$-R	TAATACGACTCACTATAGTCGAATCGTGCCCTCTGCAC/CATGGCGATATCCGGGGTGGAACGGCCATCGAG	For generating the M_b mutant expression platform RNA
EM$_C$-T7-F/AE-R	TAATACGACTCACTATATCGAATCGTGCCCTCTGCACGTCGAACGCGAGCCCCCGCG/CATGGCGATATCCGGGGTGG	For generating the M_c mutant expression platform RNA
E-T7-F/E-M10-R	TAATACGACTCACTATAGTCGAATCGTGCCCTCTGCAC/CATGGCGATTAGGGGGGTGGATCGGCCATCGAGCTTCGCGGG	For generating the M10 mutant expression platform RNA
4335-T7-F/4335-R	TAATACGACTCACTATAGGCTATGTAGCTCAGCCGG/TGGTGGCTATGGGTGGACTC	For generating tRNA XC4335 ($tRNA^{Met1}$)
4339-T7-F/4339-R	TAATACGACTCACTATAGTGCGGGGTGGAGCAGTCTGG/TGGTAGCGGGGGCAGGATTTG	For generating tRNA XC4339 ($tRNA^{fMet}$)
4381-T7-F/4381-R	TAATACGACTCACTATAGGGCCTATAGCTCAACGG/TGGTGGGCCCACCAGGATTC	For generating tRNA XC4381 ($tRNA^{Met2}$)
4339-T7-F/4339+C-R	TAATACGACTCACTATAGTGCGGGGTGGAGCAGTCTGG/GTGGTAGCGGGGGCAGGATTTGAACC	For generating tRNA XC4339 ($tRNA^{fMet}$) with 3′+C mutation
4339-T7-F/4339M$_{ant}$-R	TAATACGACTCACTATAGTGCGGGGTGGAGCAGTCTGG/TGGTAGCGGGGGCAGGATTTGAACCTGCGACCTTCGGGTTTTTAGCCC	For generating tRNA XC4339 ($tRNA^{fMet}$) with M_{anti} mutation
4339-T7-F/4339M$_{\Delta CCA}$-R	TAATACGACTCACTATAGTGCGGGGTGGAGCAGTCTGG/ACCTAGCGGGGGCAGGATTTG	For generating tRNA XC4339 ($tRNA^{fMet}$) with $M_{\Delta CCA}$ mutation
4339-T7-F/4339M$_{3'CCA}$-R	TAATACGACTCACTATAGTGCGGGGTGGAGCAGTCTGG/ACCTAGCGGGGGCAGGATTTGAACC	For generating tRNA XC4339 ($tRNA^{fMet}$) with $M_{3'CCA}$ mutation
4339-T7-F/4339-R	TAATACGACTCACTATAGTGCGGGGTGGAGCAGTCTGG/TGGTAGCGGGGGCAGGATTTG	For generating tRNA XC4339 ($tRNA^{fMet}$)
4339-T7-F/4339M$_{del}$-R	TAATACGACTCACTATAGTGCGGGGTGGAGCAGTCTGG/ACCTAGCGGGGGCAGGATTTG	For generating tRNA XC4339 ($tRNA^{fMet}$) with M_{del} mutation
4339-T7-F/4339M$_{anti}$-R	TAATACGACTCACTATAGTGCGGGGTGGAGCAGTCTGG/TGGTAGCGGGGGCAGGATTTGAACCTGCGACCTTCGGGTTTTTAGCCC	For generating tRNA XC4339 ($tRNA^{fMet}$) with M_{anti} mutation
4339-T7-F/4339M$_{3'CCA}$	TAATACGACTCACTATAGTGCGGGGTGGAGCAGTCT	For generating tRNA XC4339

-R	GG/ACCTAGCGGGGGCAGGATTTGAACC	($tRNA^{fMet}$) with $M_{3'CCA}$ mutation
4339 $M_{\Delta 5'U}$ -T7-F/4339-R	TAATACGACTCACTATAGGCGGGGTGGAGCAGTCTGG/TGGTAGCGGGGGCAGGATTTG	For generating tRNA XC4339 ($tRNA^{fMet}$) with $M_{\Delta 5'U}$ mutation
4339-T7-F/4339M_{7374}-R	TAATACGACTCACTATAGTGCGGGGTGGAGCAGTCTGG/TGGATGCGGGGGCAGGATTTG	For generating tRNA XC4339 ($tRNA^{fMet}$) with M_{7374} mutation
4339 M_{D-loop} -T7-F/4339-R	TAATACGACTCACTATAGTGCGGGGTGGAGCAGTAAAGCAGCTCGTCGGG/TGGTAGCGGGGGCAGGATTTG	For generating tRNA XC4339 ($tRNA^{fMet}$) with M_{D-loop} mutation
4339-T7-F/4339M_{T-loop}-R	TAATACGACTCACTATAGTGCGGGGTGGAGCAGTCTGG/TGGTAGCGGGGGCAGGATAACAACCTGCGACCTTCGGG	For generating tRNA XC4339 ($tRNA^{fMet}$) with M_{T-loop} mutation
4333-T7-F/4333R	TAATACGACTCACTATAGCGCTCGTAGCTCAGCCGG/TGGTGCGCCCGGAGGGATTC	For generating tRNA XC4333($tRNA^{Arg}$)
4334-T7-F/4334R	TAATACGACTCACTATAGTGCCCCGTCGCCAAGCGG/TGGCTGCCCCGGATGGATTC	For generating tRNA XC4338 ($tRNA^{Leu}$)
4338-T7-F/4338R	TAATACGACTCACTATAGCCCAGGTGGCGGAATTGG/TGGTGCCCAAGAGGGGACTC	For generating tRNA XC4334 ($tRNA^{Gln}$)
4363-T7-F/4363R	TAATACGACTCACTATAGCCCAGGTGGCGGAATTGG/TGGTGCCCAGGAGAGGACTC	For generating tRNA XC4363 ($tRNA^{Leu}$)
4371-T7-F/4371R	TAATACGACTCACTATAGGCGAAAGTGGCGGAATTGG/TGGTGCGAAAGGGGGGACTC	For generating tRNA XC4371 ($tRNA^{Leu}$)
4341-T7-F/4341R	TAATACGACTCACTATAGGGGCGGTTAGCTCAGCGG/TGGTGGGCGGTACAGGGTTC	For generating tRNA XC4341 ($tRNA^{Val}$)
4342-T7-F/4342R	TAATACGACTCACTATAGCGGGGTATAGCGCAGCCTGG/TGGTCGGGGTAGCCGGATTC	For generating tRNA XC4342 ($tRNA^{Pro}$)
4345-T7-F/4345R	TAATACGACTCACTATAGCGGGGTGTAGCTCAGTCTGG/TGGTCGGGGAGACAGGATTC	For generating tRNA XC4345 ($tRNA^{Pro}$)
4375-T7-F/4375R	TAATACGACTCACTATAGCGGGGTATAGCGCAGTCTGG/TGGTCGGGGTAGAGGGATTC	For generating tRNA XC4375 ($tRNA^{Pro}$)
4344-T7-F/4344R	TAATACGACTCACTATAGGGGGCGGTAGCTCAGCTGGG/TGGTGGAGCGGAAGGGGATC	For generating tRNA XC4344 ($tRNA^{Ala}$)
4348-T7-F/4348R	TAATACGACTCACTATAGGGGGCCATAGCTCAGCTGGG/TGGTGGAGCCAGGCGGGATC	For generating tRNA XC4348 ($tRNA^{Ala}$)
4385-T7-F/4385R	TAATACGACTCACTATAGGGGGCCTTAGCTCAGCTGGG/TGGTGGAGCCTGTCGGGATC	For generating tRNA XC4385 ($tRNA^{Ala}$)
4392-T7-F/4392R	TAATACGACTCACTATAGGGGGCCTTAGCTCAGCTGGG/TGGTGGAGCCTGTCGGGATC	For generating tRNA XC4392 ($tRNA^{Ala}$)
4359-T7-F/4359R	TAATACGACTCACTATAGGGAGCGATGCCCGAGCGG/TGGCGGAGCGAGAGGGATTC	For generating tRNA XC4359 ($tRNA^{Ser}$)
4360-T7-F/4360R	TAATACGACTCACTATAGGGAGAGATGGCCGAGCGG/TGGCGGAGAGAGTGGGATTC	For generating tRNA XC4360 ($tRNA^{Ser}$)
4366-T7-F/	TAATACGACTCACTATAGGGAGAGGTGTCCGAGTG	For generating tRNA XC4366

4366R	G/TGGCGGAGAGAGGGGGATTC	(tRNA^{Ser})
4367-T7-F/<br>4367R	TAATACGACTCACTATAGGGAGAGGTGGCAGAGCG<br>G/ATTCGAACCCCCGAGGCGC	For generating tRNA XC4367 (tRNA^{Ser})
4364-T7-F/<br>4364R	TAATACGACTCACTATAGGCCGGAATAGCTCAGTTG<br>G/TGGTGCCGGAAATAGGAATC	For generating tRNA XC4364 (tRNA^{Thr})
4377-T7-F/<br>4377R	TAATACGACTCACTATAGGGGCTCACGTAGCTCAGT<br>CGG/TGGTGCTCACGAAAGGAATC	For generating tRNA XC4377 (tRNA^{Thr})
Plac-SAM-I-F/SAM-I-M1-R	CCCGAATTCGGCTTTACACTTTATGCTTCCGGCTCG<br>TATGTTGTGTGGAACCATCGAGACCGGCGGAGGG/C<br>ATGGCGATATCCGGGGTGGATCGCGGTACGAGCTTC<br>GCGGGAGCTCGCG	M1 SAM-I$_{Xcc}$-coding fragment with *lac* promoter
Plac-SAM-I-F/SAM-I-M2-R	CCCGAATTCGGCTTTACACTTTATGCTTCCGGCTCG<br>TATGTTGTGTGGAACCATCGAGACCGGCGGAGGG/C<br>ATGGCGATATCCGGGGTCCTACGGCCATCGAGCTTC<br>GCGGGAGCTCGCG	M2 SAM-I$_{Xcc}$-coding fragment with *lac* promoter
Plac-SAM-I-F/SAM-I-M(1+2)-R	CCCGAATTCGGCTTTACACTTTATGCTTCCGGCTCG<br>TATGTTGTGTGGAACCATCGAGACCGGCGGAGGG/C<br>ATGGCGATATCCGGGGTCCTACGCGGTACGAGCTTC<br>GCGGGAGCTCGCG	M(1+2) SAM-I$_{Xcc}$-coding fragment with *lac* promoter
Plac-SAM-I-Ma1-F/<br>SAM-I-R	CCC***GAATTC***GGCTTTACACTTTATGCTTCCGGCTCGT<br>ATGTTGTGTGGAACCTACGAGACCGGCGGAGGG/C<br>ATGGCGATATCCGGGGTGG	Ma1SAM-I$_{Xcc}$-coding fragment with *lac* promoter
Plac-SAM-I-Ma2-F/<br>SAM-I-R	CCC***GAATTC***GGCTTTACACTTTATGCTTCCGGCTCGT<br>ATGTTGTGTGGAACCATCGGAACCGGCGGAGGG/CA<br>TGGCGATATCCGGGGTGG	Ma2 SAM-I$_{Xcc}$-coding fragment with *lac* promoter
SD^{+}-*gusA*-F/*gusA*-R	CCCCGGATATCGCCATGATTGAGGAGTCCCTTATG<br>TTACGTCCTGTAGAAACCCC/CCCAAGCTTTCATTG<br>TTTGCCTCCCTGCTGCGG	*gus*A coding sequence with SD sequence and 20 bp SAM-I$_{Xcc}$ sequence
p0-F/*gusA*-R	CCCGAATTCGGCTTTACACTTTATGCTTCCGGCTCG<br>TATGTTGTGTGGATGAGGAGTCCCTTATGTTACGTC<br>CTGTAGAAACCCC/CCCAAGCTTTCATTGTTTGCCT<br>CCCTGCTGCGG	For generating the *lac* promoter -SD^{+}*gusA* fusion fragment
pTrp-F/*gusA*-R	CCCGAATTCGGCTTTACACTTTATGCTTCCGGCTCG<br>TATGTTGTGTGGACAGATACCCAGCCCGCCTAATGA<br>GCGGGCTTTTTTTTTGAGGAGTCCCTTATGTTACGT<br>CCTGTAGAAACCCC/CCCAAGCTTTCATTGTTTGCC<br>TCCCTGCTGCGG	For generating the *lac* promoter –trp terminator-SD^{+}*gusA* fusion fragment
P5-F/*gusA*-R	CCCGAATTCGGCTTTACACTTTATGCTTCCGGCTCG<br>TATGTTGTGTGGATCGAATCGTGCCCTCTGCACGTC<br>GATGAGGAGTCCCTTATGTTACGTCCTGTAGAAACC<br>CC/CCCAAGCTTTCATTGTTTGCCTCCCTGCTGCGG	For generating the *lac* promoter –hairpin P5 -SD^{+}*gusA* fusion fragment
P6-F/*gusA*-R	CCCGAATTCGGCTTTACACTTTATGCTTCCGGCTCG<br>TATGTTGTGTGGACGAGCTCCCGCGAAGCTCGTGA<br>GGAGTCCCTTATGTTACGTCCTGTAGAAACCCC/CCC	For generating the *lac* promoter –hairpin P6 -SD^{+}*gusA* fusion fragment

	AAGCTTTCATTGTTTGCCTCCCTGCTGCGG	
P7-F/*gusA*-R	CCCGAATTCGGCTTTACACTTTATGCTTCCGGCTCGTATGTTGTGTGGAATGGCCGATCCACCCCGGATATCGCCATGTGAGGAGTCCCTTATGTTACGTCCTGTAGAAACCCC/CCCAAGCTTTCATTGTTTGCCTCCCTGCTGCGG	For generating the *lac* promoter –hairpin P7 -SD$^+$*gusA* fusion fragment
P56-F/*gusA*-R	CCCGAATTCGGCTTTACACTTTATGCTTCCGGCTCGTATGTTGTGTGGATCGAATCGTGCCCTCTGCACGTCGAACGCGAGCTCCCGCGAAGCTCGTGAGGAGTCCCTTATGTTACGTCCTGTAGAAACCCC/CCCAAGCTTTCATTGTTTGCCTCCCTGCTGCGG	For generating the *lac* promoter –hairpin P5+P6 -SD$^+$*gusA* fusion fragment
P67-F/*gusA*-R	CCCGAATTCGGCTTTACACTTTATGCTTCCGGCTCGTATGTTGTGTGGACGAGCTCCCGCGAAGCTCGATGGCCGATCCACCCCGGATATCGCCATGTGAGGAGTCCCTTATGTTACGTCCTGTAGAAACCCC/CCCAAGCTTTCATTGTTTGCCTCCCTGCTGCGG	For generating the *lac* promoter –hairpin P6+P7-SD$^+$*gusA*fusion fragment
P567-F/*gusA*-R	CCCGAATTCGGCTTTACACTTTATGCTTCCGGCTCGTATGTTGTGTGGATCGAATCGTGCCCTCTGCACGTCGAACGCGAGCTCCCGCGAAGCTCGATGGCCGATCCACCCCGGATATCGCCATGTGAGGAGTCCCTTATGTTACGTCCTGTAGAAACCCC/CCCAAGCTTTCATTGTTTGCCTCCCTGCTGCGG	For generating the *lac* promoter –hairpin P5+P6+P7-SD$^+$*gusA*fusion fragment
OE4335-F/OE4335-R	CCCGAATTCGGCTATGTAGCTCAGCCGGTTAG/CCCAAGCTTTGGTGGCTATGGGTGGACTC	Elongator tRNA (tRNA^{Met}) coding sequence
OE4339-F/OE4339-R	CCCGAATTCTGCGGGGTGGAGCAGTCTGG/CCCAAGCTTTGGTAGCGGGGGCAGGATTTG	Initiator tRNA (tRNA^{fMet}) coding sequence
OE4339-F/OE4339M-R	CCCGAATTCTGCGGGGTGGAGCAGTCTGG/CCCAAGCTTTGGATGCGGGGGCAGGATTTG	Coding sequence for the initiator tRNA (tRNA^{fMet}) mutant with the U73A74 → A73U74 substitution
Oligo-1	ATATCCGGGGTG	For RNase H cleavage experiment
Oligo-2	GCCATCGAGC	For RNase H cleavage experiment

* Underlined nucleotides indicate the T7 promoter sequence. Red and green nucleotides refer to the restriction sites and the *lac* promoter sequence, respectively.

Supplementary Table 3 | A summary of the tRNA genes in the genome of *Xcc* strain 8004[29].

#	Gene name	Product	Start	End	Strand	Length (bp)
1	*XC4333*	$tRNA^{Arg}$	465776	465852	+	77
2	*XC4334*	$tRNA^{Gln}$	580608	580682	-	75
3	*XC4335*	$tRNA^{Met}$	580737	580813	-	77
4	*XC4336*	$tRNA^{Gly}$	1230865	1230938	+	74
5	*XC4337*	$tRNA^{Lys}$	1387670	1387745	+	76
6	*XC4338*	$tRNA^{Leu}$	1908307	1908391	+	85
7	*XC4339*	$tRNA^{Met}$	1923546	1923622	+	77
8	*XC4340*	$tRNA^{Asn}$	1952406	1952482	+	77
9	*XC4341*	$tRNA^{Val}$	1955335	1955409	+	75
10	*XC4342*	$tRNA^{Pro}$	1993172	1993248	+	77
11	*XC4343*	$tRNA^{Phe}$	2022474	2022549	+	76
12	*XC4344*	$tRNA^{Ala}$	2194951	2195026	-	76
13	*XC4345*	$tRNA^{Pro}$	2278418	2278494	-	77
14	*XC4346*	$tRNA^{Leu}$	2373049	2373135	+	87
15	*XC4347*	$tRNA^{Glu}$	2373877	2373952	-	76
16	*XC4348*	$tRNA^{Ala}$	2374006	2374081	-	76
17	*XC4349*	$tRNA^{Glu}$	2374835	2374910	-	76
18	*XC4350*	$tRNA^{Ala}$	2374958	2375033	-	76
19	*XC4351*	$tRNA^{Gly}$	2414130	2414205	-	76
20	*XC4352*	$tRNA^{Cys}$	2414286	2414359	-	74
21	*XC4353*	$tRNA^{Gly}$	2414429	2414504	-	76
22	*XC4354*	$tRNA^{Leu}$	2515193	2515276	-	84
23	*XC4355*	$tRNA^{Arg}$	2899430	2899506	-	77
24	*XC4356*	$tRNA^{Glu}$	2987981	2988055	+	75
25	*XC4357*	$tRNA^{Arg}$	3028285	3028361	+	77
26	*XC4358*	$tRNA^{Arg}$	3028458	3028534	+	77
27	*XC4359*	$tRNA^{Ser}$	3035112	3035204	-	93
28	*XC4360*	$tRNA^{Ser}$	3177001	3177090	-	90
29	*XC4361*	$tRNA^{Leu}$	3267563	3267649	+	87
30	*XC4363*	$tRNA^{Leu}$	3408796	3408882	+	87
31	*XC4364*	$tRNA^{Thr}$	3695224	3695299	-	76
32	*XC4365*	$tRNA^{Val}$	3866283	3866357	-	75
33	*XC4366*	$tRNA^{Ser}$	3890211	3890303	-	93
34	*XC4367*	$tRNA^{Ser}$	3896458	3896532	-	75
35	*XC4368*	$tRNA^{Asp}$	3904897	3904973	-	77
36	*XC4369*	$tRNA^{Asp}$	3905070	3905146	-	77
37	*XC4370*	$tRNA^{Val}$	3905176	3905250	-	75
38	*XC4371*	$tRNA^{Leu}$	3911992	3912076	-	85
39	*XC4372*	$tRNA^{Lys}$	3912154	3912229	-	76
40	*XC4373*	$tRNA^{His}$	3912313	3912389	-	77

41	*XC4374*	$tRNA^{Arg}$	3912421	3912497	-	77
42	*XC4375*	$tRNA^{Pro}$	3912541	3912617	-	77
43	*XC4376*	$tRNA^{Trp}$	4002575	4002650	-	76
44	*XC4377*	$tRNA^{Thr}$	4003994	4004069	-	76
45	*XC4378*	$tRNA^{Gly}$	4004103	4004176	-	74
46	*XC4379*	$tRNA^{Tyr}$	4004206	4004291	-	86
47	*XC4380*	$tRNA^{Gln}$	4008125	4008201	-	77
48	*XC4381*	$tRNA^{Met}$	4498738	4498813	-	76
49	*XC4384*	$tRNA^{Ile}$	4635692	4635768	-	77
50	*XC4385*	$tRNA^{Ala}$	4635788	4635863	-	76
51	*XC4387*	$tRNA^{Xaa}$	4686060	4686127	+	68
52	*XC4388*	$tRNA^{Thr}$	4738454	4738529	-	76
53	*XC4391*	$tRNA^{Ile}$	5023079	5023155	-	77
54	*XC4392*	$tRNA^{Ala}$	5023175	5023250	-	76

Supplementary Table 4 | The species and strains relative to the GenBank accession number present in Supplementary Fig. 24.

#	Accession number	Strain	#	Accession number	Strain	#	Accession number	Strain
1	CP000050.1	*Xcc* strain 8004	17	CP008989.1	*X. citri* subsp. *citri* strain UI7	33	CP013679.1	*X. oryzae* pv. *oryzae* strain
2	AE008922.1	*Xcc* ATCC 33913	18	CP008995.1	*X. citri* subsp. *citri* strain NT17	34	CP013666.1	*X. oryzae* pv. *oryzae* strain AXO1947
3	CP011946.1	*Xcc* strain 17	19	CP006857.1	*X. citri* subsp. *citri* A306	35	CP013678.1	*X. oryzae* pv. *oryzae* strain PXO563
4	CP021146.1	*Xcc* strain ICMP 4013	20	FO681494.1	*X. fuscans* subsp. *fuscans* strain4834-R	36	CP000967.2	*X. oryzae* pv. *oryzae* PXO99A
5	AM920689.1	*Xcc* strain B100	21	AE008923.1	*X. axonopodis* pv. *citri* strain 306	37	AP008229.1	*X. oryzae* pv. *Oryzae* MAFF 311018
6	CP018731.1	*X. gardneri* strain ICMP 7383	22	CP014347.1	*X. axonopodis* pv. *eieffenbachiae* LMG 695	38	AE013598.1	*X. oryzae* pv. *Oryzae* KACC 10331
7	CP0128728.1	*X. gardneri* strain JS749-3	23	CP018475.1	*X. perforans* strain LH3	39	CP011962.1	*X. oryzae* pv. *Oryzicola* strain CFBP2286
8	CP011256.1	*X. campestris* strain 17	24	CP018467.1	*X. euvesicatoria* strain LMG930	40	CP011961.1	*X. oryzae* pv. *Oryzicola* strain RS105
9	CP012251.1	*X. arboricola* pv. *juglandis* strain Xaj 417	25	CO017190.1	*X. campestris* pv. *vesicatoria* strain 85-10	41	CP011960.1	*X. oryzae* pv. *oryzicola* strain L8
10	CP018725.1	*X. vesicatoria* ATCC35937 strain LMG 911	26	CP002914.1	*X. axonopodis* pv. *citrumelo* F1	42	CP011958.1	*X. oryzae* pv. *oryzicola* strain CFBP7331
11	CP018470.1	*X. vesicatoria* strain LM159	27	AM039952.1	*X. campestris* pv. *vesicatoria*	43	CP011956.1	*X. oryzae* pv. *Oryzicola* strain BLS279
12	CP018858.1	*X. citri* pv. *citri* strain LH2019	28	CP022270.1	*X. citri* pv. *vignicola* strain CFBP7113	44	CP003057.2	*X. oryzae* pv. *oryzicola* BLS256
13	CP018850.1	*X. citri* pv. *citri* strain LJ207-7	29	CP017020.1	*X. citri* pv. *malvacearum* strain MSCT	45	LT604072.1	*X. translucens* pv. *translucens* DSM 18974 isolate peng1

14	CO018847.1	*X. citri* pv. *citri* strain LL074-4	30	CP016833.1	*X. fragariae* isolate Fap29	46	CP008714.1	*X. translucens* pv. *undulosa* strain Xtu 4699
15	CP017188.1	*X. axonopodis* pv. *glycines* strain 8ra	31	CP008989.1	*X. citri* subsp. *citri* strain UI7	47	CP010409.1	*X. sacchari* strain R1
16	CP011827.2	*X. citri* pv. *citri*strainjx-6	32	CP016830.1	*X. fragariae* isolate Fap21	48	FP565176.1	*X. albilineans* GPE PC73

The full scan figure for the western blots

Fig.5b

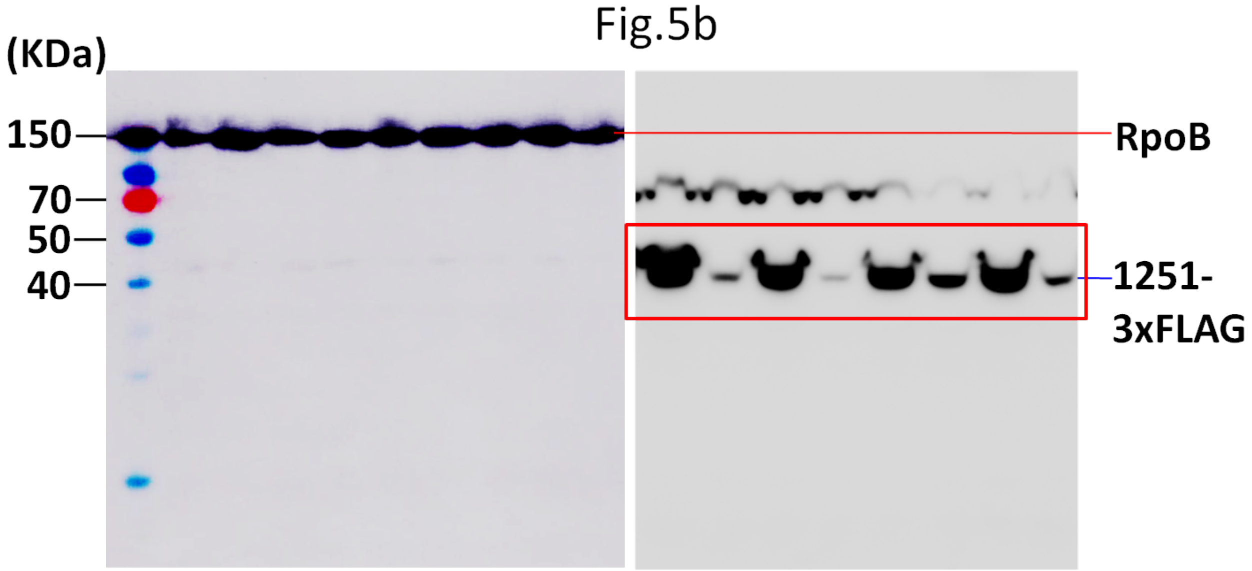

Supplementary Fig.23(b)

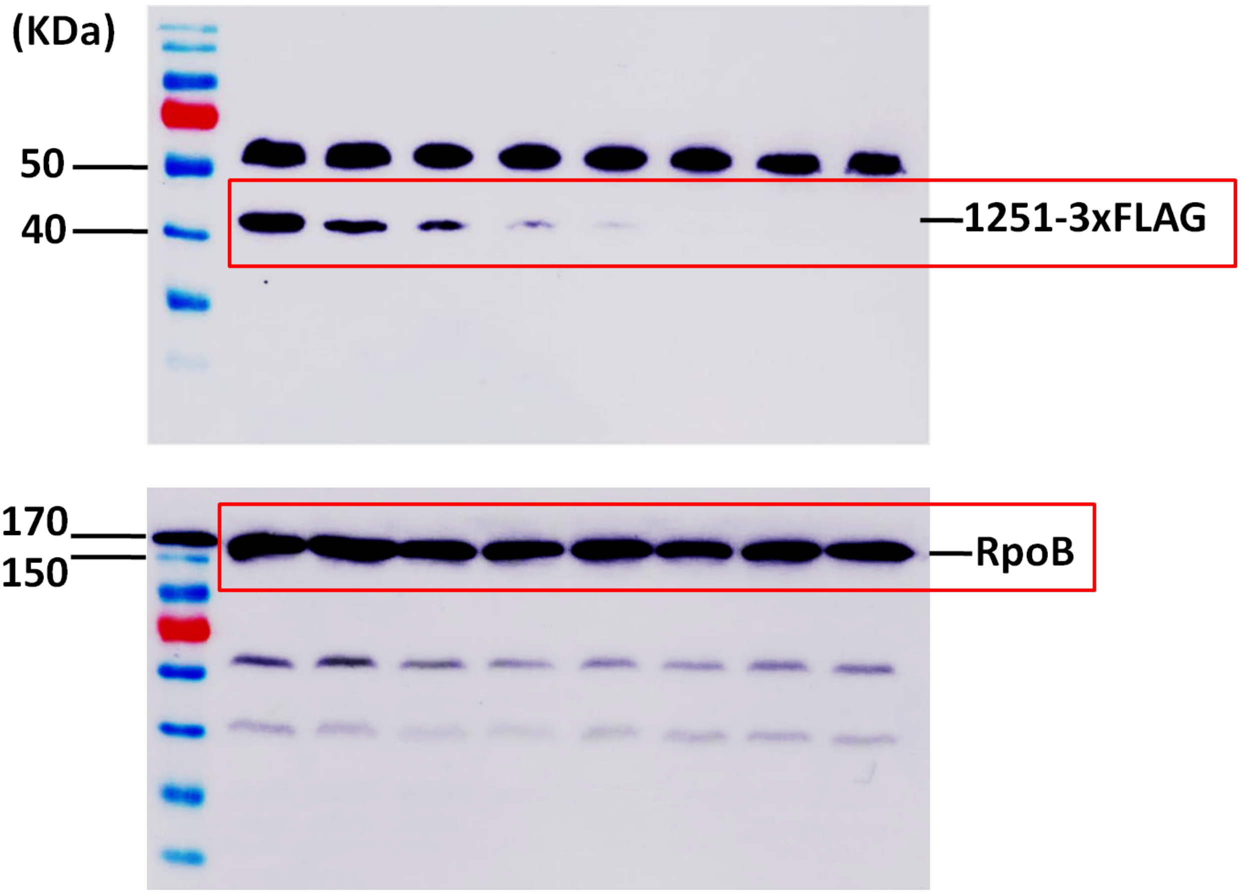

Supplementary References

49. Yanisch-Perron, C. *et al*. Improved M13 phage cloning vectors and host strains: nucleotide sequences of the M13mp18 and pUC19 vectors. *Gene* **33**, 103-119 (1985).

50. Huynh, T. V. *et al*. Bacterial blight of soybean: regulation of a pathogen gene determining host cultivar specificity. *Science* **245**, 1374-1377 (1989).

51. Sukchawalit, R. *et al*. Construction and characterization of regulated L-arabinose-inducible broad host range expression vectors in *Xanthomonas*. *FEMS Microbiol. Lett*. **181**, 217-223 (1999).
