# Supplementary material for: A SAM-I riboswitch with the ability to sense and respond to uncharged initiator tRNA
Source: Nat Commun. 2020 Jun 3;11:2794. doi: 10.1038/s41467-020-16417-z (PMC7270179; doi:10.1038/s41467-020-16417-z)
Supplement: Supplementary file 3 — Reporting Summary [file 41467_2020_16417_MOESM3_ESM.pdf]

nature research

Corresponding author(s): Ji-Liang Tang

Last updated by author(s): Apr 8, 2020

Reporting Summary

Nature Research wishes to improve the reproducibility of the work that we publish. This form provides structure for consistency and transparency in reporting. For further information on Nature Research policies, see Authors & Referees and the Editorial Policy Checklist.

Statistics

For all statistical analyses, confirm that the following items are present in the figure legend, table legend, main text, or Methods section.

n/a	Confirmed	
☒	☐	The exact sample size (*n*) for each experimental group/condition, given as a discrete number and unit of measurement
☒	☐	A statement on whether measurements were taken from distinct samples or whether the same sample was measured repeatedly
☐	☒	The statistical test(s) used AND whether they are one- or two-sided<br>*Only common tests should be described solely by name; describe more complex techniques in the Methods section.*
☒	☐	A description of all covariates tested
☒	☐	A description of any assumptions or corrections, such as tests of normality and adjustment for multiple comparisons
☐	☒	A full description of the statistical parameters including central tendency (e.g. means) or other basic estimates (e.g. regression coefficient) AND variation (e.g. standard deviation) or associated estimates of uncertainty (e.g. confidence intervals)
☐	☒	For null hypothesis testing, the test statistic (e.g. *F*, *t*, *r*) with confidence intervals, effect sizes, degrees of freedom and *P* value noted<br>*Give P values as exact values whenever suitable.*
☒	☐	For Bayesian analysis, information on the choice of priors and Markov chain Monte Carlo settings
☒	☐	For hierarchical and complex designs, identification of the appropriate level for tests and full reporting of outcomes
☒	☐	Estimates of effect sizes (e.g. Cohen's *d*, Pearson's *r*), indicating how they were calculated

Our web collection on statistics for biologists contains articles on many of the points above.

Software and code

Policy information about availability of computer code

Data collection	No software was used.
Data analysis	No software was used.

For manuscripts utilizing custom algorithms or software that are central to the research but not yet described in published literature, software must be made available to editors/reviewers. We strongly encourage code deposition in a community repository (e.g. GitHub). See the Nature Research guidelines for submitting code & software for further information.

Data

Policy information about availability of data

All manuscripts must include a data availability statement. This statement should provide the following information, where applicable:

- Accession codes, unique identifiers, or web links for publicly available datasets
- A list of figures that have associated raw data
- A description of any restrictions on data availability

All data that support the findings of this study are available from the corresponding authors upon request. The source data underlying Figs 1a, d, 2c, 3b, 4c, d, e, and 5b, c and Supplementary Figs 3, 4b, 5-9, 11, 12, 16-22, 23a, b and 25b are provided as a Source Data file.

Field-specific reporting

Please select the one below that is the best fit for your research. If you are not sure, read the appropriate sections before making your selection.

☒ Life sciences ☐ Behavioural & social sciences ☐ Ecological, evolutionary & environmental sciences

Life sciences study design

All studies must disclose on these points even when the disclosure is negative.

Sample size	In this work, we study a single-celled organism (Bacteria), no sample-sized calculation was performed.
Data exclusions	No data were excluded.
Replication	All attempts at replication were successful.
Randomization	In this work, we study a single-celled organism (Bacteria), sample randomization is not relevant to our study.
Blinding	In this work, we study a single-celled organism (Bacteria), blinding is not relevant to our study.

Reporting for specific materials, systems and methods

We require information from authors about some types of materials, experimental systems and methods used in many studies. Here, indicate whether each material, system or method listed is relevant to your study. If you are not sure if a list item applies to your research, read the appropriate section before selecting a response.

Materials & experimental systems

n/a	Involved in the study	
☐	☒	Antibodies
☒	☐	Eukaryotic cell lines
☒	☐	Palaeontology
☒	☐	Animals and other organisms
☒	☐	Human research participants
☒	☐	Clinical data

Methods

n/a	Involved in the study	
☒	☐	ChIP-seq
☒	☐	Flow cytometry
☒	☐	MRI-based neuroimaging

Antibodies

Antibodies used	1. Anti-FLAG M2 monoclonal antibody (Supplier: Beyotime, China/Catalog number: AF519)<br>2. Horseradish peroxidase-linked anti-mouse secondary antibody (Supplier: Beyotime, China/Catalog number: A0208)<br>3. Anti-RNA polymerase beta (Supplier: Abcam, Shanghai, China/Catalog number: ab191598/Clone name: Rabbit monoclonal [EPR18704] to RNA polymerase beta)
Validation	Host species: Rabbit/Mouse/Goat. All antibodies used in this study are for research use only, not for use in diagnostic procedures.
