# Supplementary figures and images for: A SAM-I riboswitch with the ability to sense and respond to uncharged initiator tRNA
Source: Nat Commun. 2020 Jun 3;11:2794. doi: 10.1038/s41467-020-16417-z (PMC7270179; doi:10.1038/s41467-020-16417-z)

Fig.3b

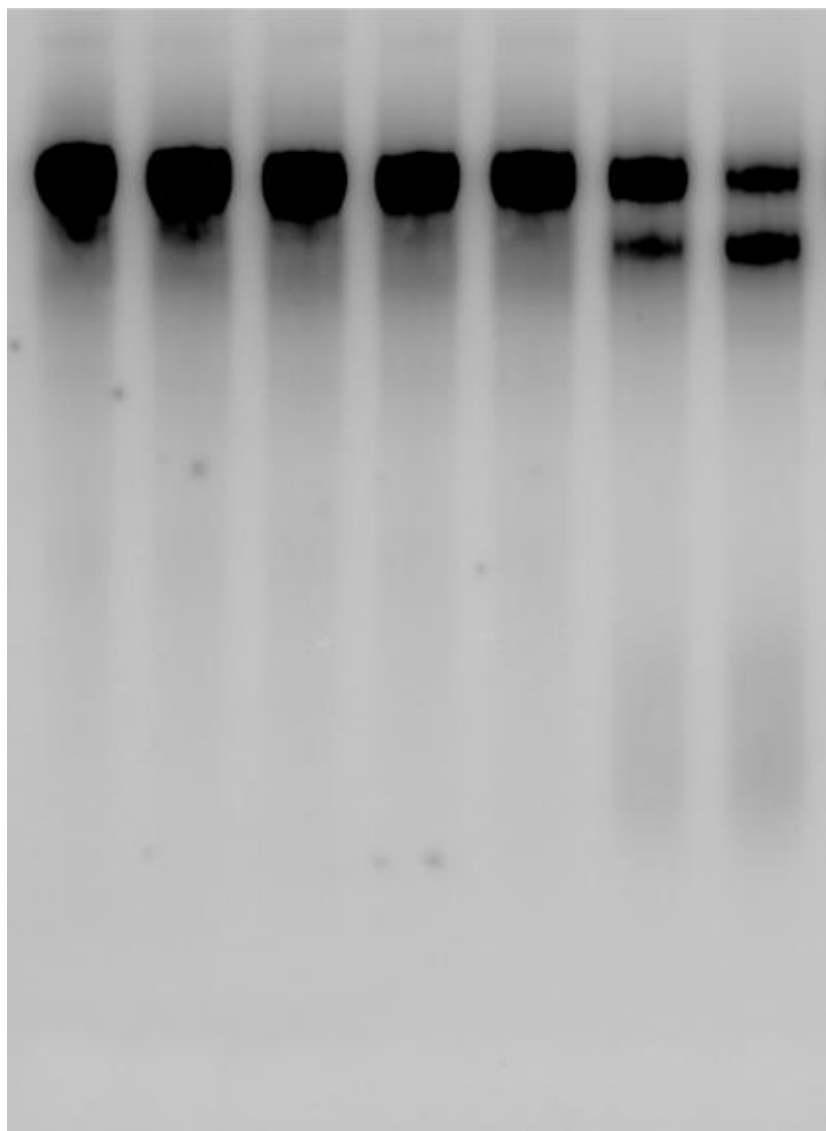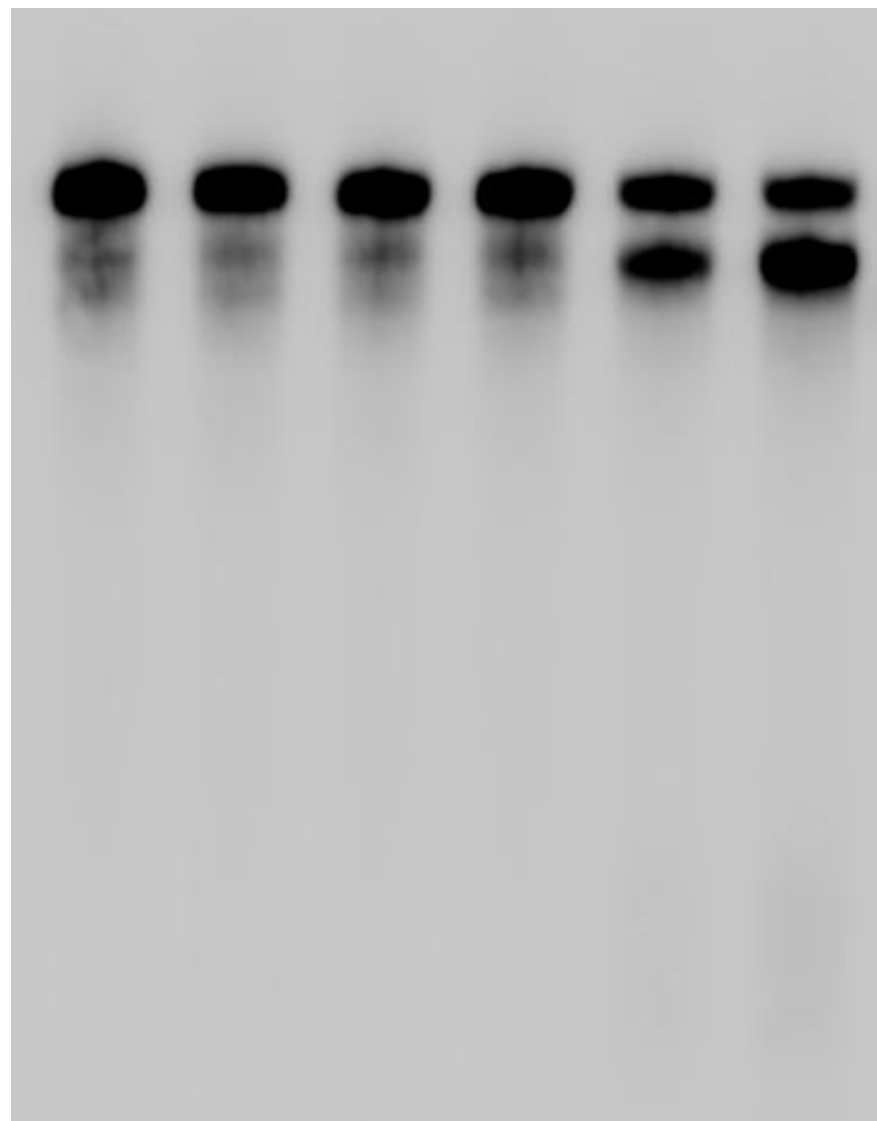

Supplement: Supplementary file 4 — Source Data [file 41467_2020_16417_MOESM4_ESM.zip › Tang et al. Source Data file/the Source Data of Fig.3(b).pdf]

Fig.4c

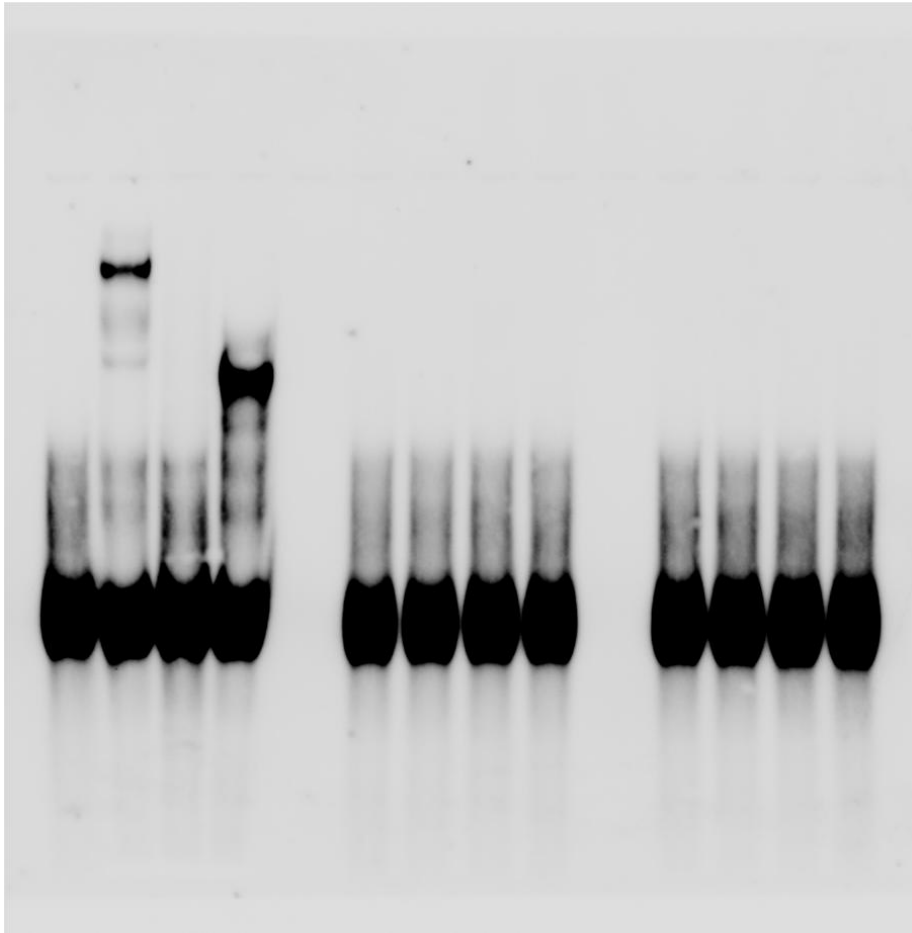

Fig.4d

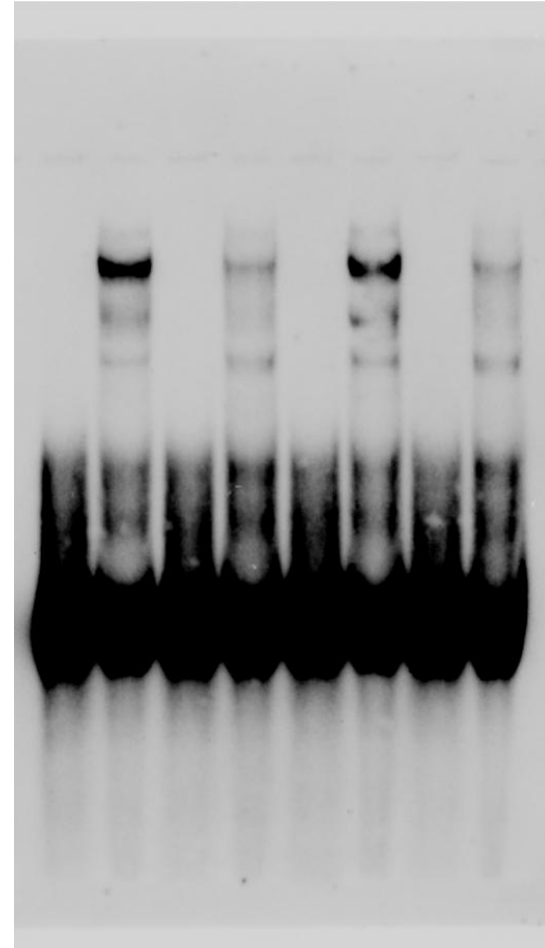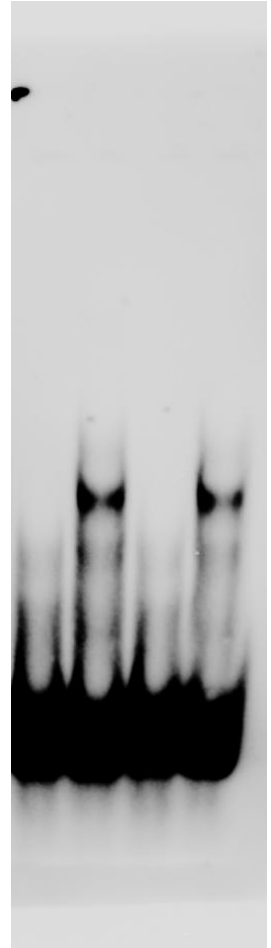

Fig.4e

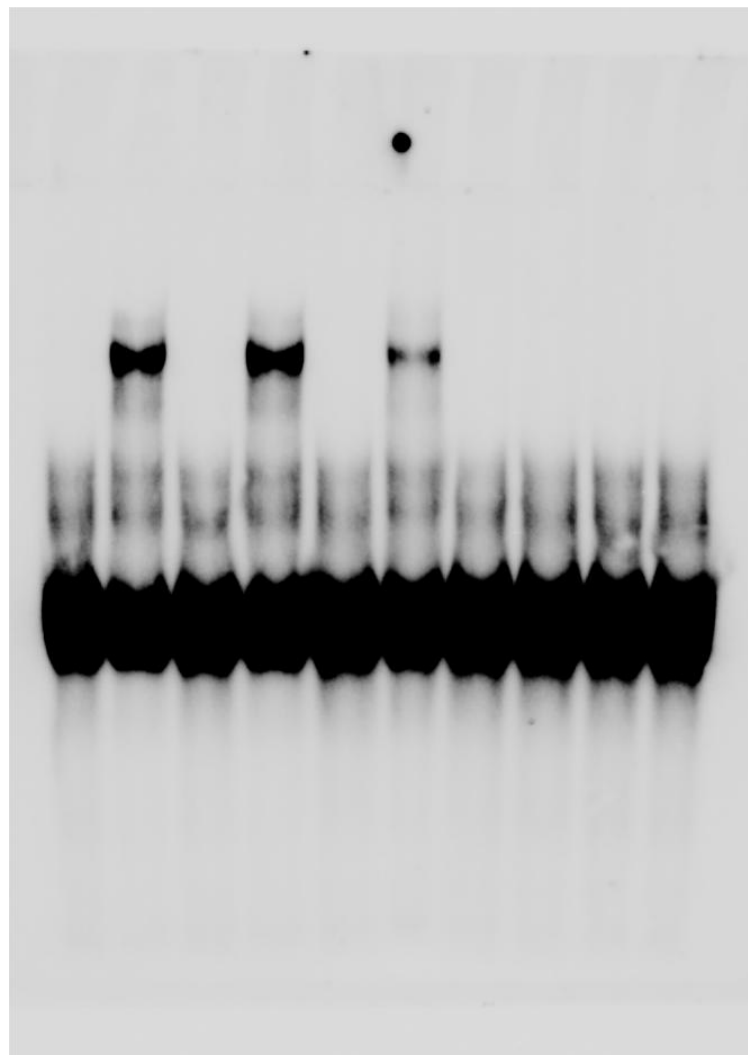

Supplement: Supplementary file 4 — Source Data [file 41467_2020_16417_MOESM4_ESM.zip › Tang et al. Source Data file/the Source Data of Fig.4(c,d,e).pdf]

Fig.5b

(KDa)

150  
70  
50  
40

RpoB

1251-  
3xFLAG

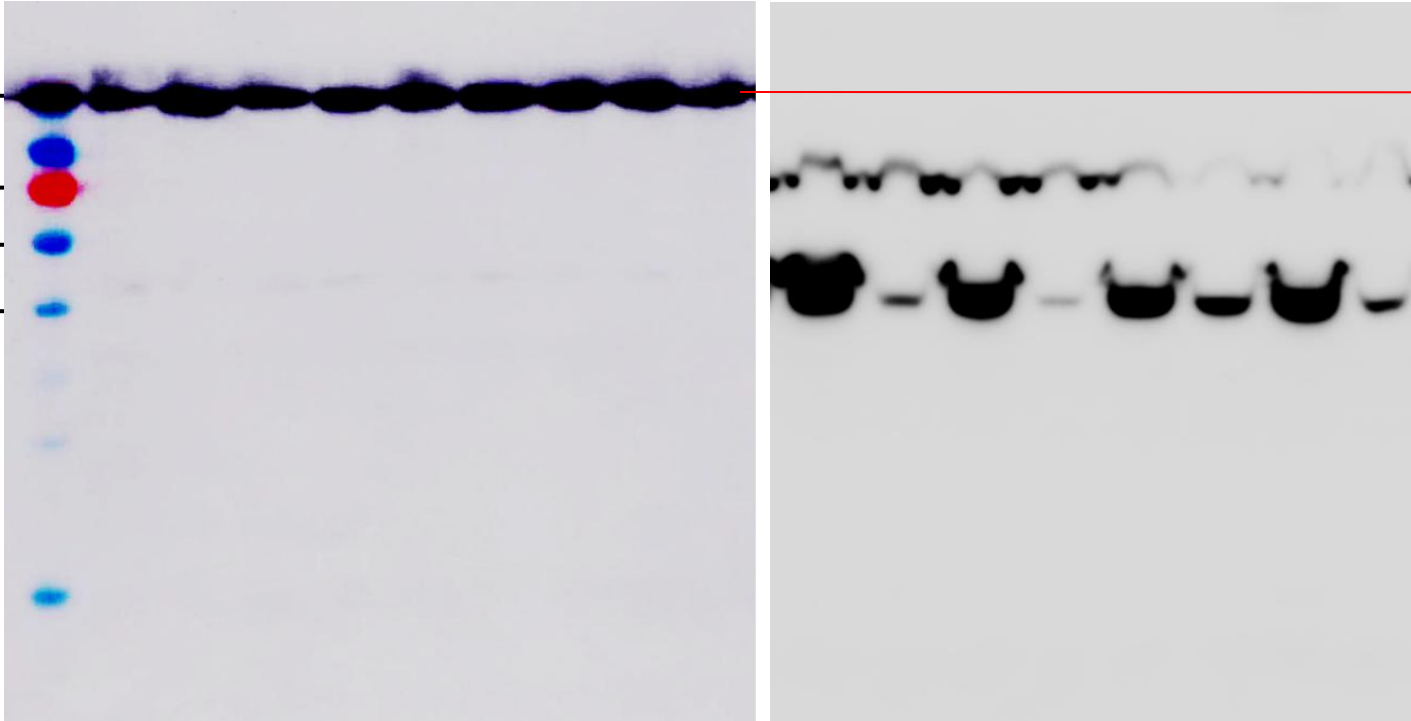

Supplement: Supplementary file 4 — Source Data [file 41467_2020_16417_MOESM4_ESM.zip › Tang et al. Source Data file/the Source Data of Fig.5(b).pdf]

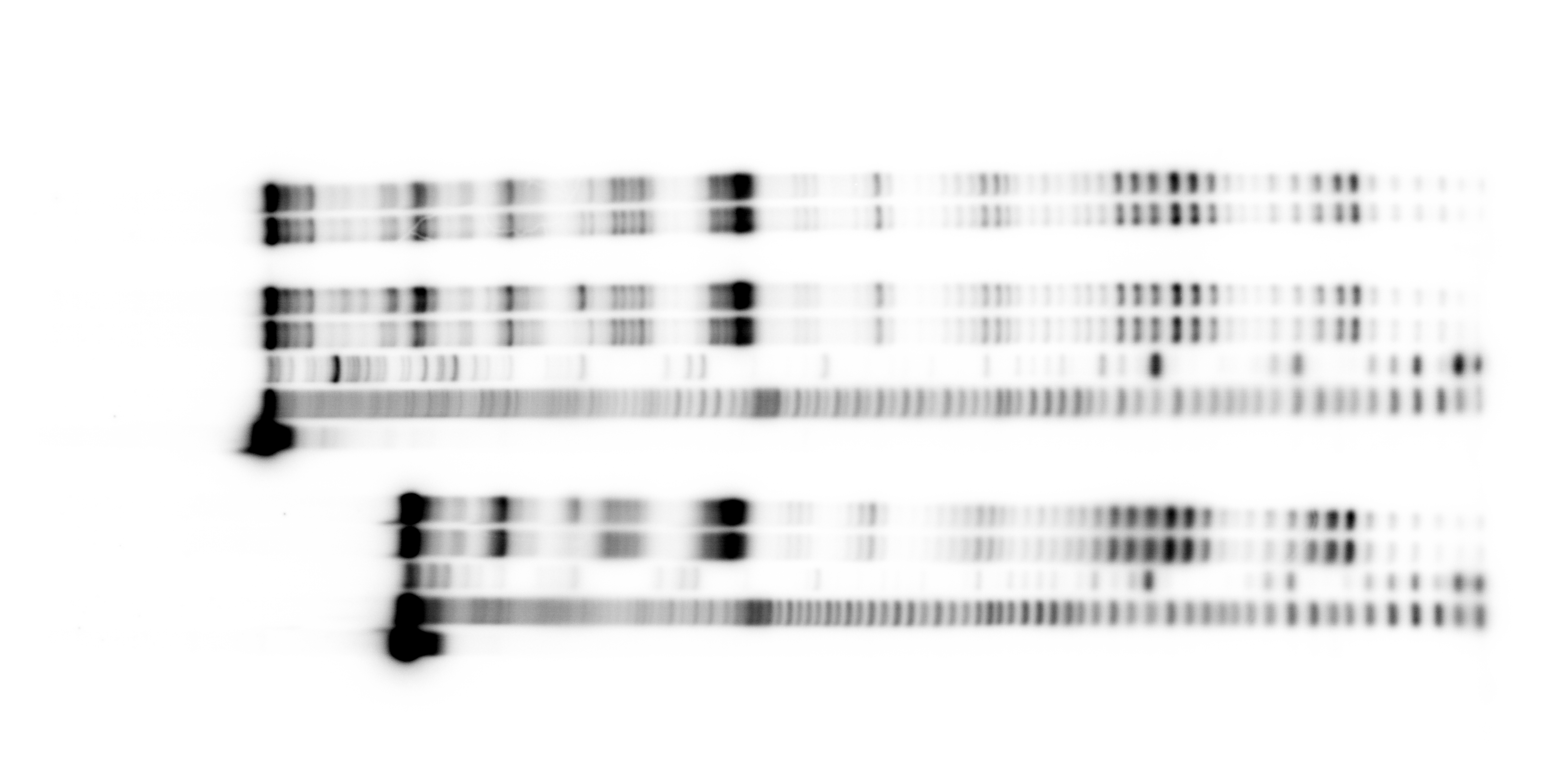

Supplement: Supplementary file 4 — Source Data [file 41467_2020_16417_MOESM4_ESM.zip › Tang et al. Source Data file/the Source Data of Figure1(d).tif]

Supplementary Fig.12b

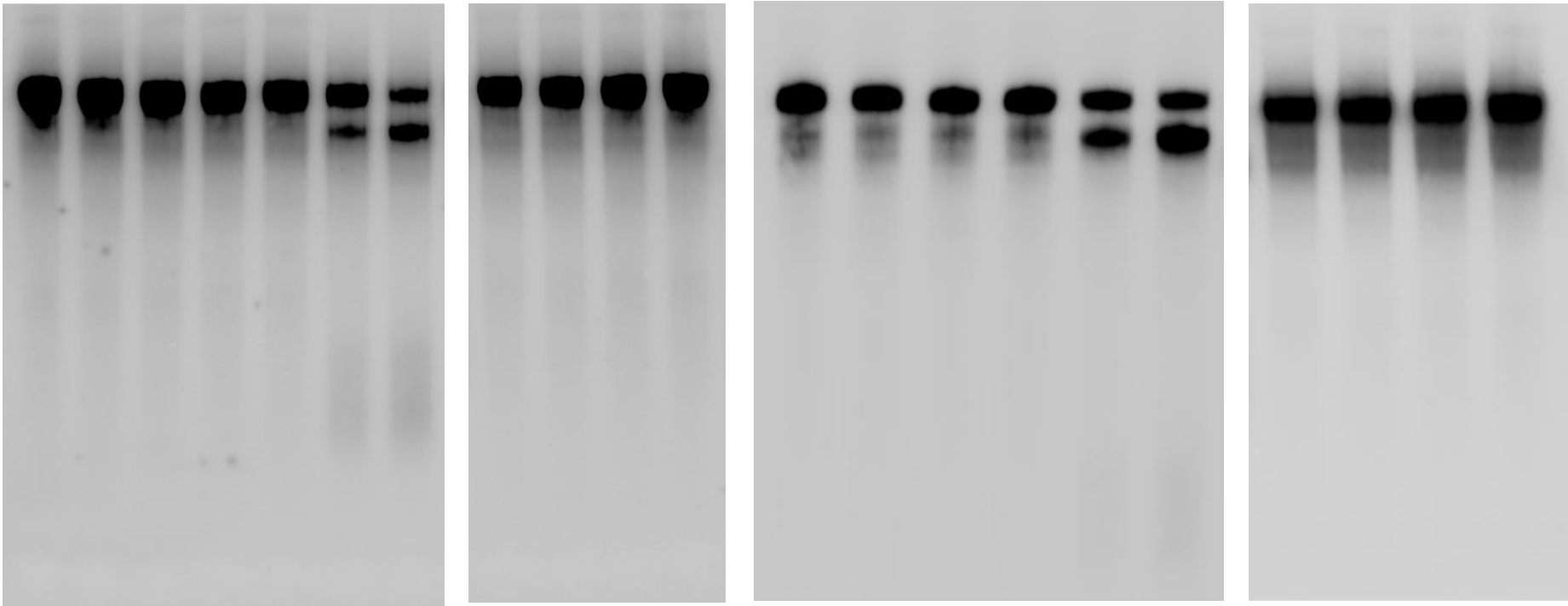

Supplement: Supplementary file 4 — Source Data [file 41467_2020_16417_MOESM4_ESM.zip › Tang et al. Source Data file/the Source Data of Supplementary Fig.12(b).pdf]

Supplementary Fig.16

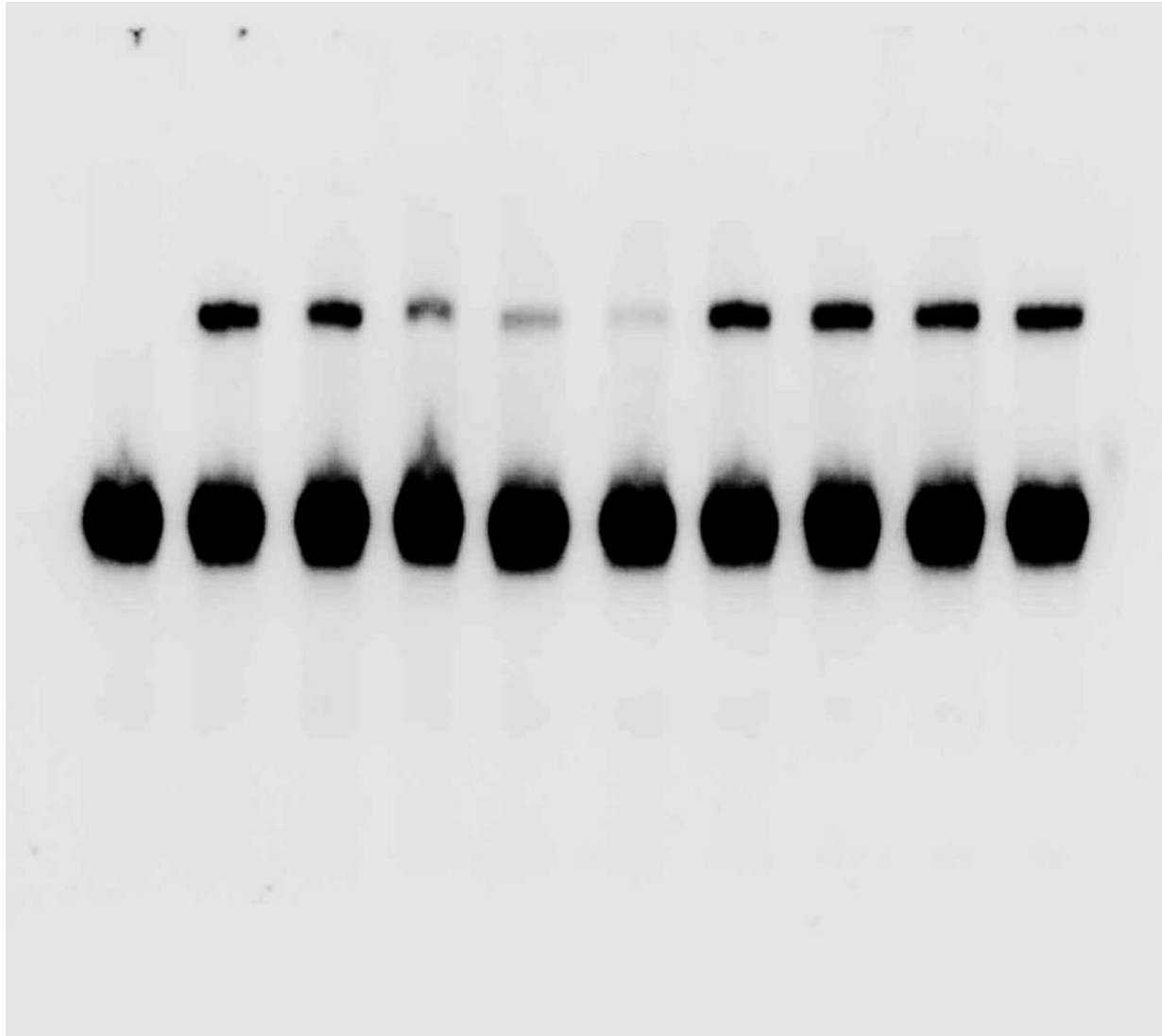

Supplement: Supplementary file 4 — Source Data [file 41467_2020_16417_MOESM4_ESM.zip › Tang et al. Source Data file/the Source Data of Supplementary Fig.16.pdf]

Supplementary Fig.17

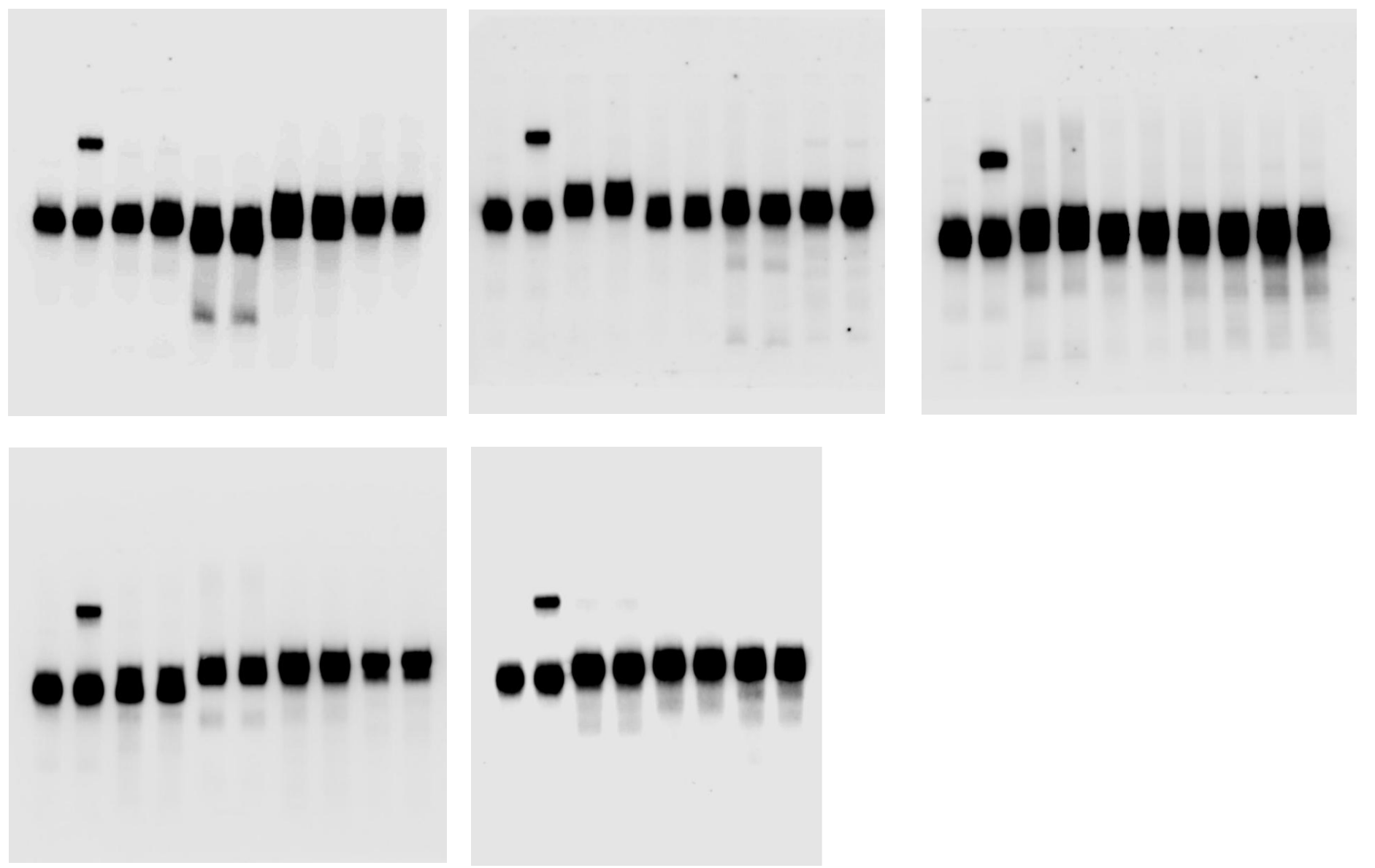

Supplement: Supplementary file 4 — Source Data [file 41467_2020_16417_MOESM4_ESM.zip › Tang et al. Source Data file/the Source Data of Supplementary Fig.17.pdf]

Supplementary Fig.18b

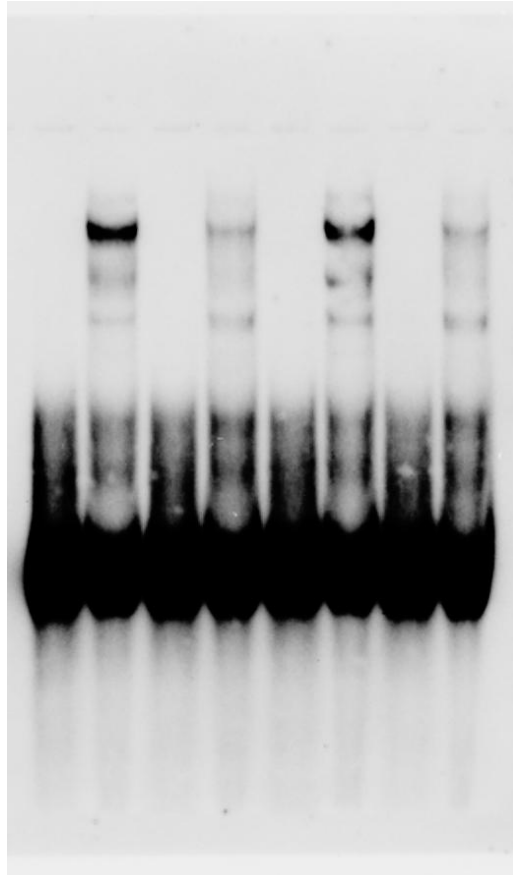

2017.01.23

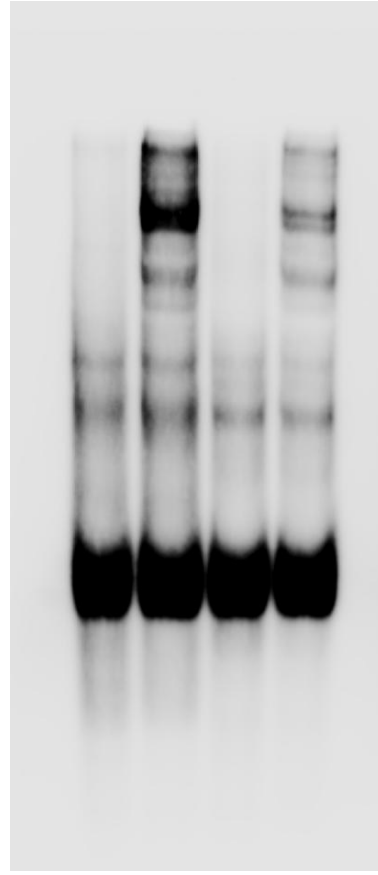

2015.02.02

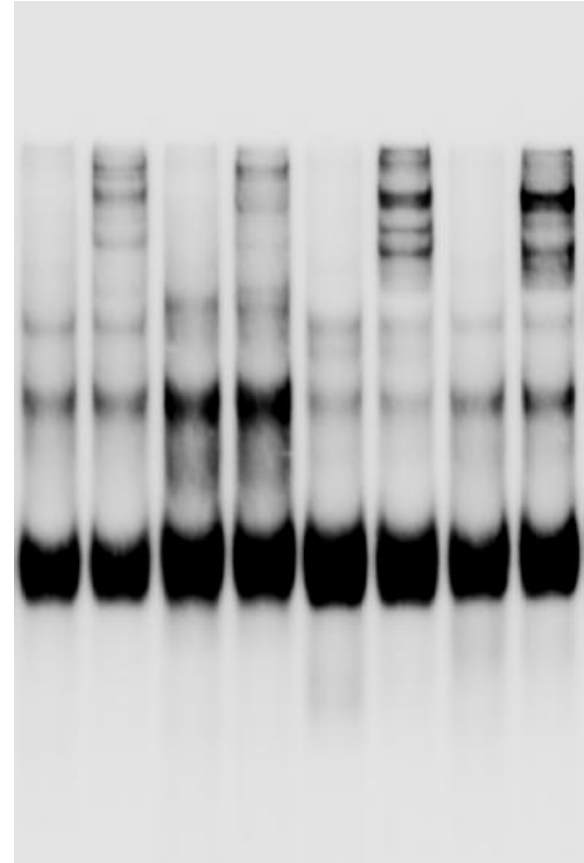

2015.02.12

Supplementary Fig.18d

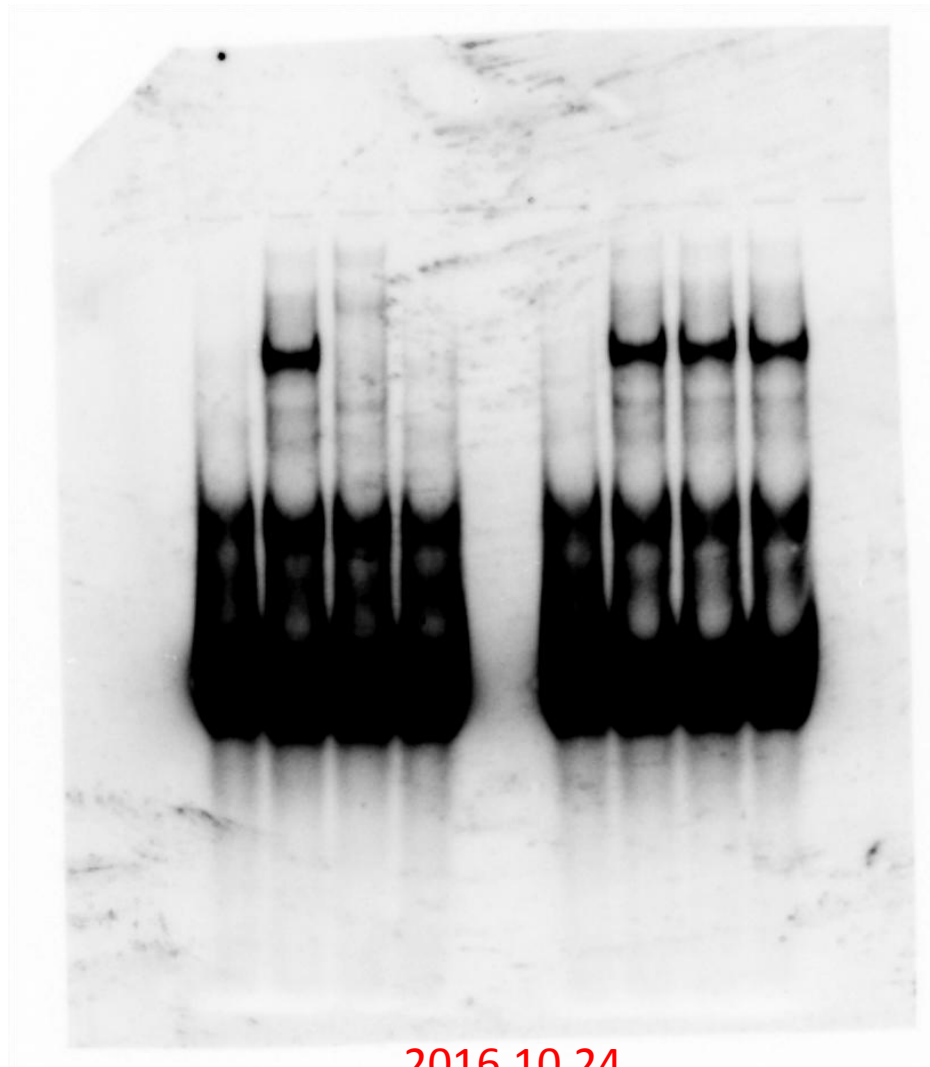

2016.10.24

Supplement: Supplementary file 4 — Source Data [file 41467_2020_16417_MOESM4_ESM.zip › Tang et al. Source Data file/the Source Data of Supplementary Fig.18.pdf]

Supplementary Fig.19

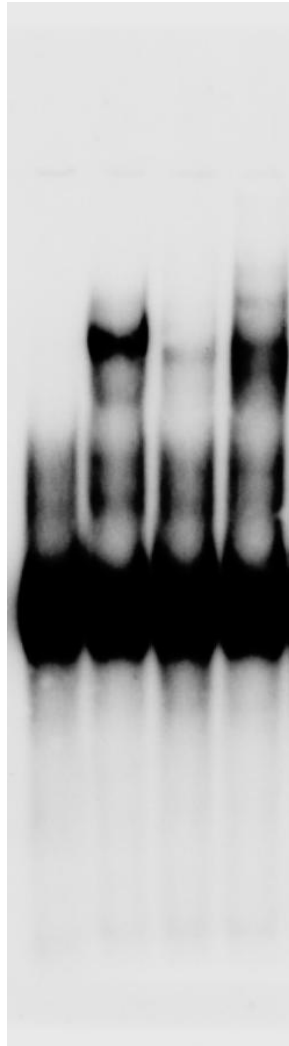

Supplement: Supplementary file 4 — Source Data [file 41467_2020_16417_MOESM4_ESM.zip › Tang et al. Source Data file/the Source Data of Supplementary Fig.19.pdf]

Supplementary Fig.20

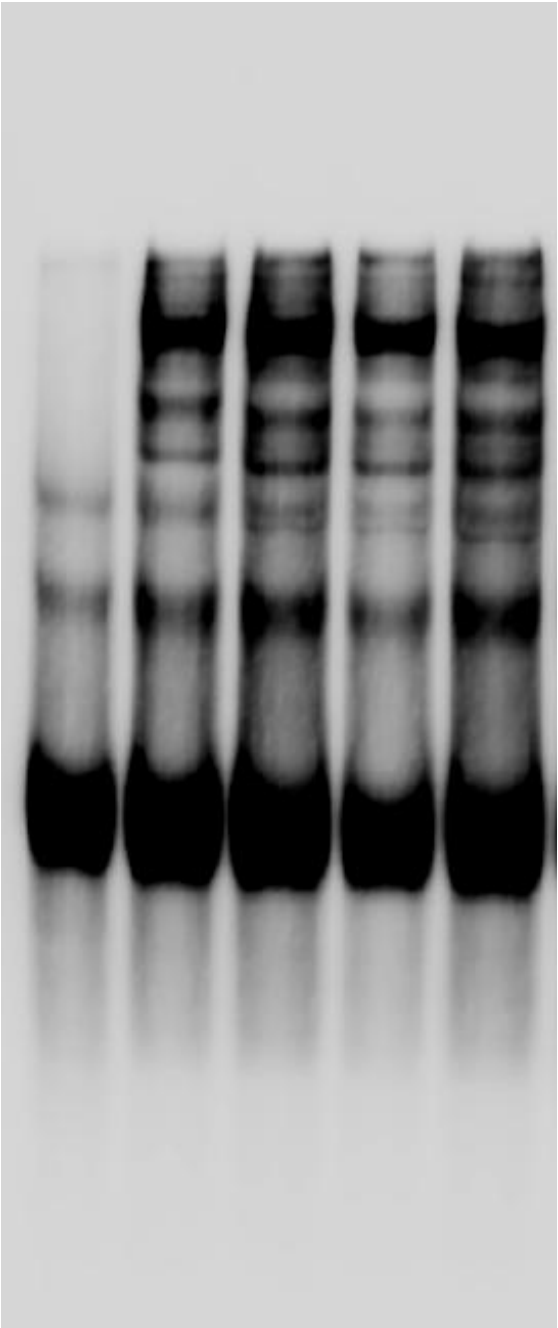

Supplement: Supplementary file 4 — Source Data [file 41467_2020_16417_MOESM4_ESM.zip › Tang et al. Source Data file/the Source Data of Supplementary Fig.20.pdf]

Supplementary Fig.21

**4339 probe**

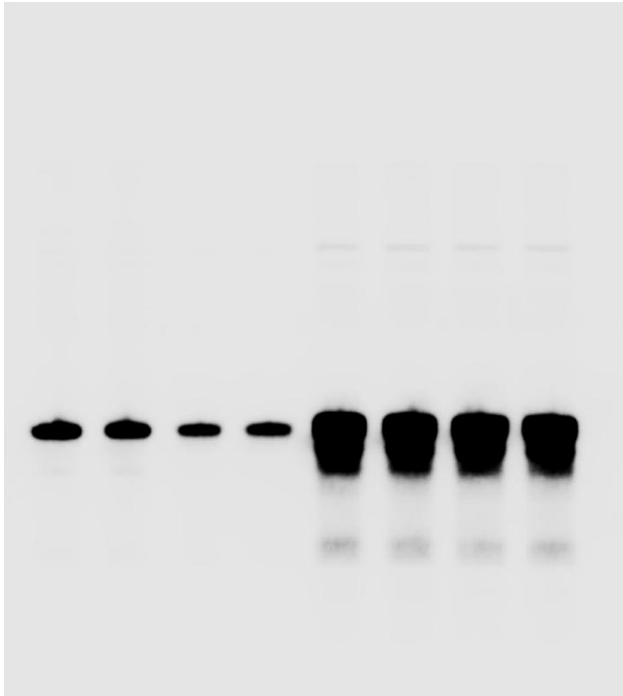

**4335 probe**

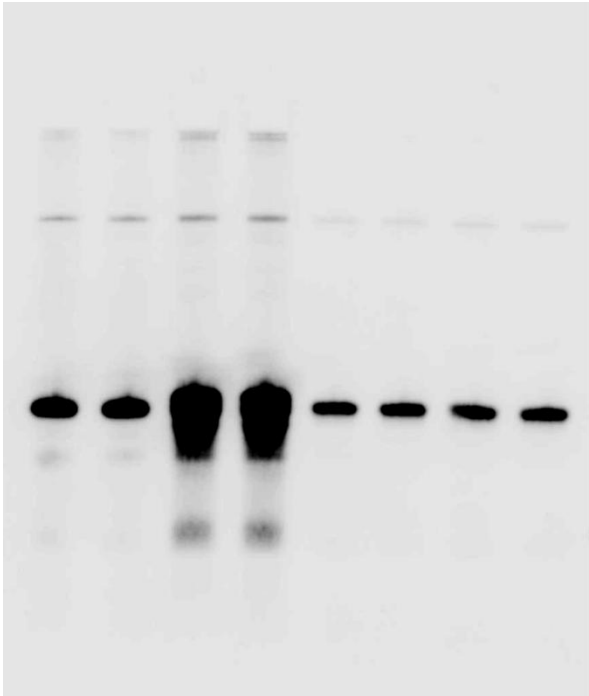

**5S rRNA probe**

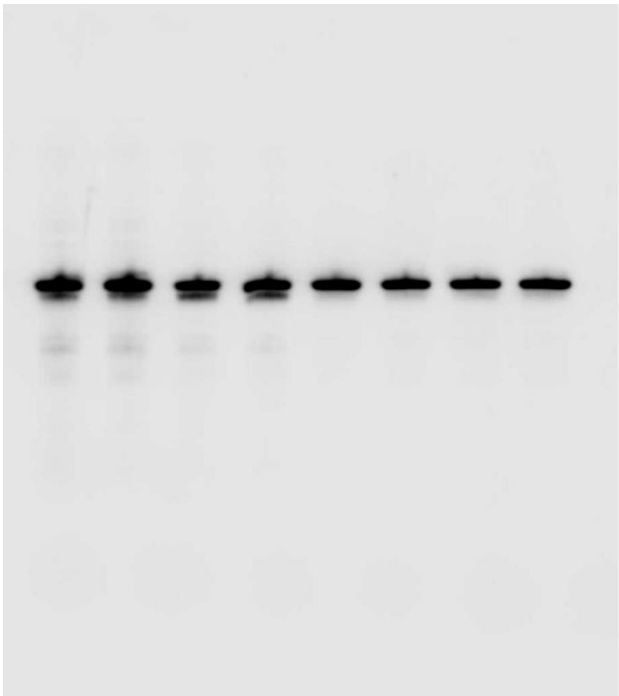

Supplement: Supplementary file 4 — Source Data [file 41467_2020_16417_MOESM4_ESM.zip › Tang et al. Source Data file/the Source Data of Supplementary Fig.21.pdf]

Supplementary Fig.22

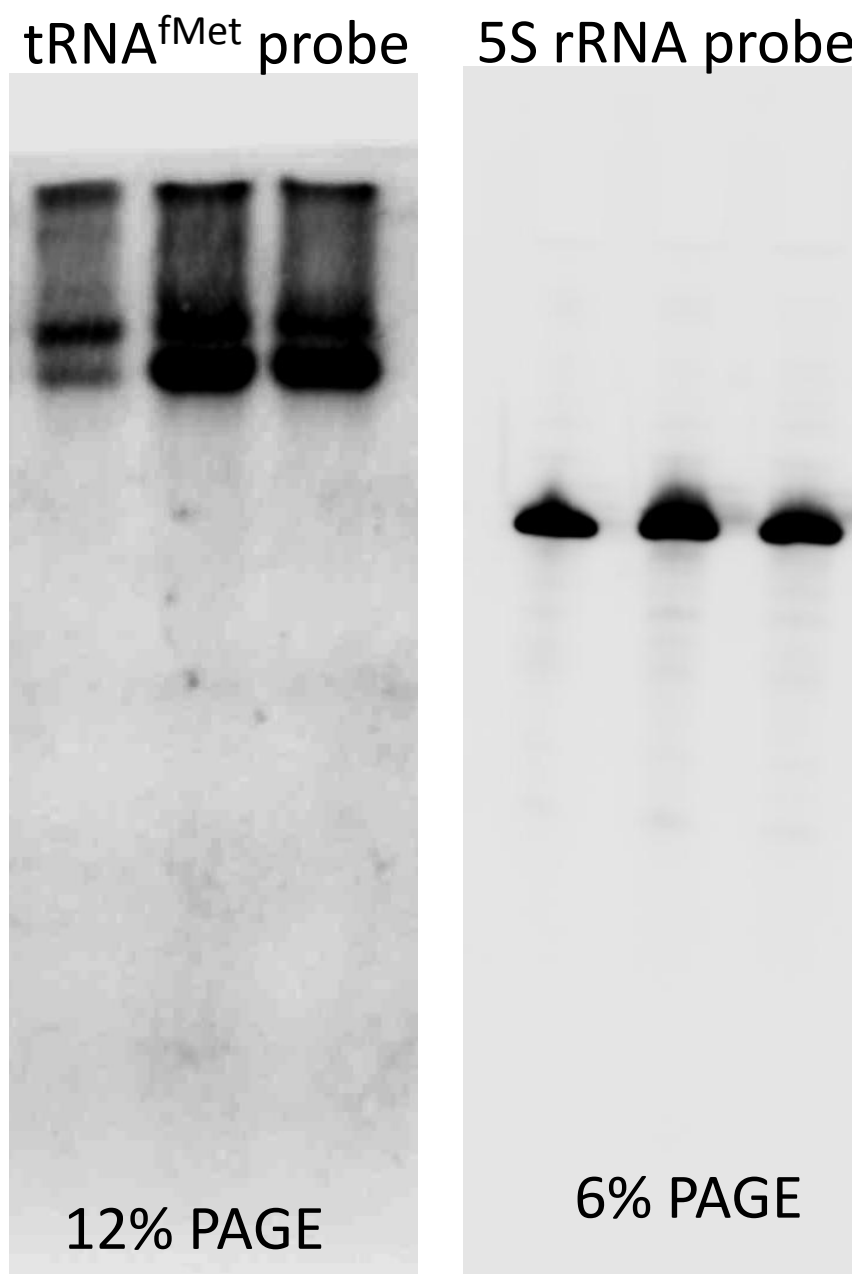

Supplement: Supplementary file 4 — Source Data [file 41467_2020_16417_MOESM4_ESM.zip › Tang et al. Source Data file/the Source Data of Supplementary Fig.22.pdf]

Supplementary Fig.23(b)

(KDa)

50

40

1251-3xFLAG

170

150

RpoB

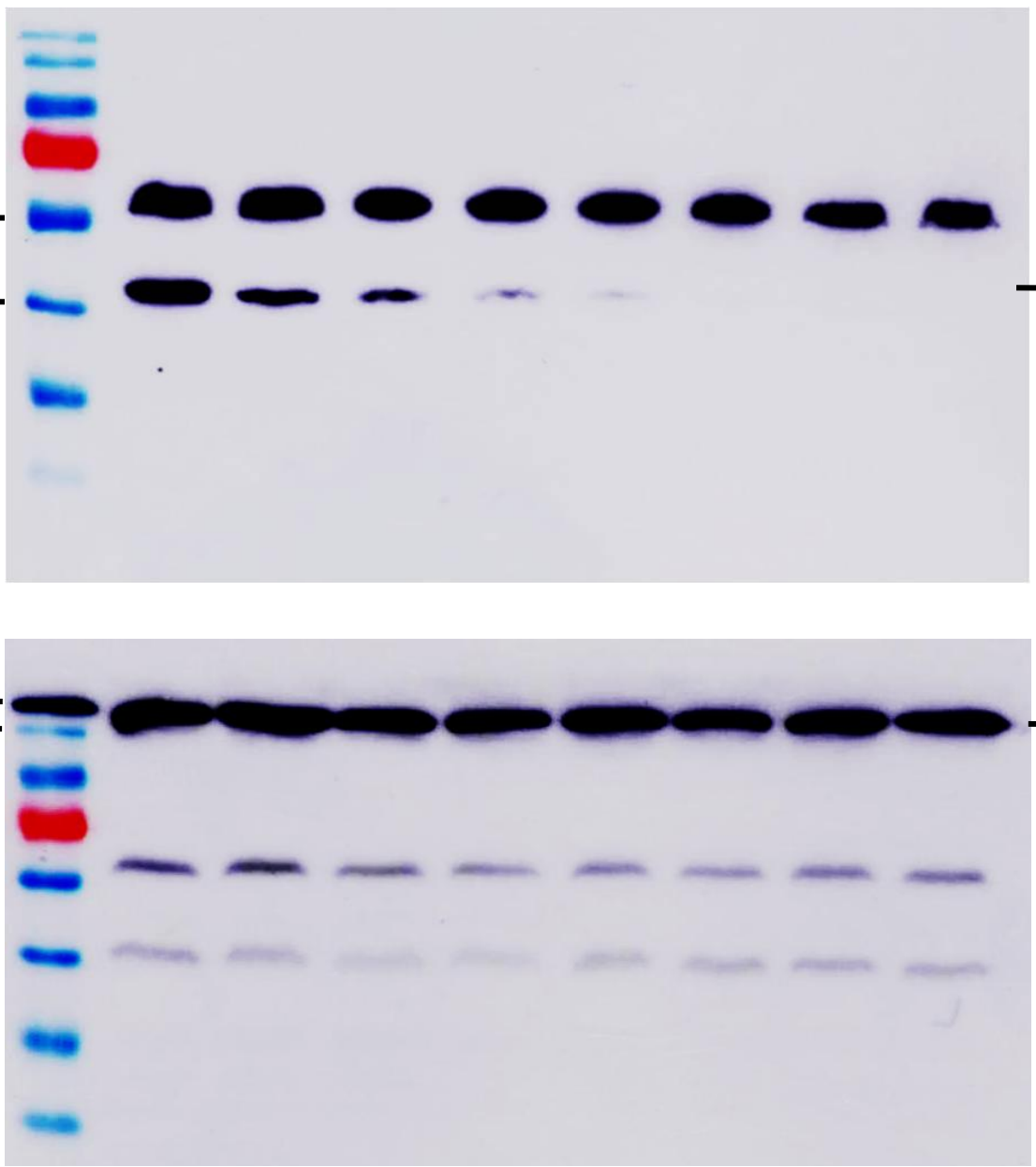

Supplement: Supplementary file 4 — Source Data [file 41467_2020_16417_MOESM4_ESM.zip › Tang et al. Source Data file/the Source Data of Supplementary Fig.23(b).pdf]

Supplementary Fig.25(b)

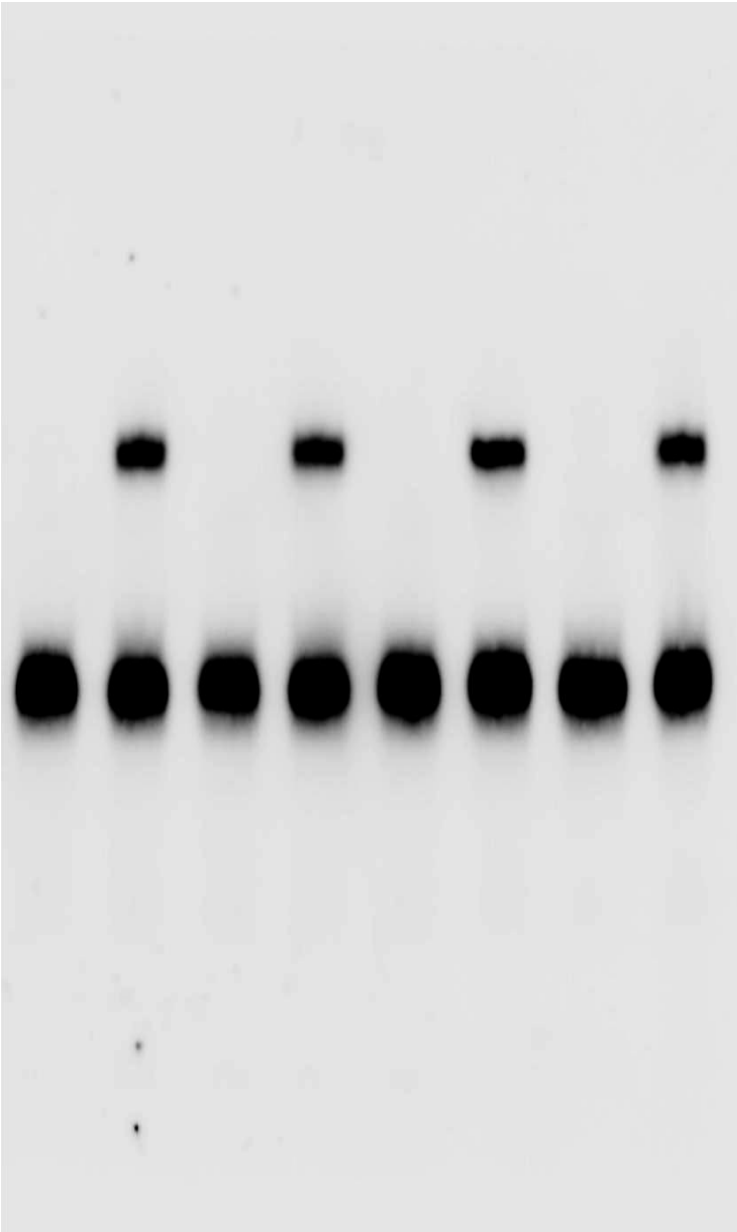

Supplement: Supplementary file 4 — Source Data [file 41467_2020_16417_MOESM4_ESM.zip › Tang et al. Source Data file/the Source Data of Supplementary Fig.25(b).pdf]

Supplementary Fig. 3

Repeat-I

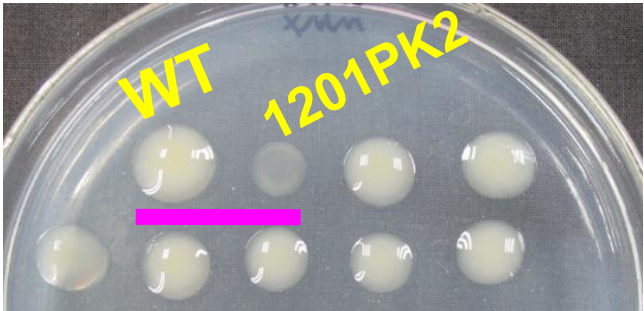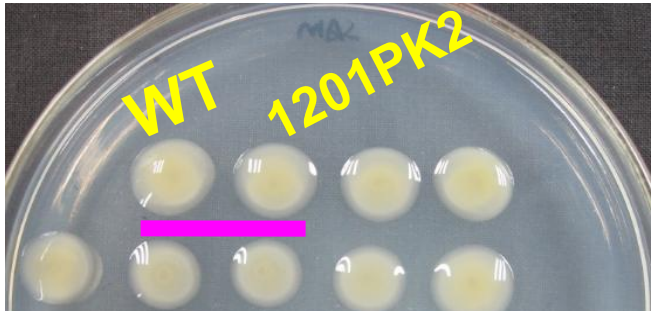

Repeat-II

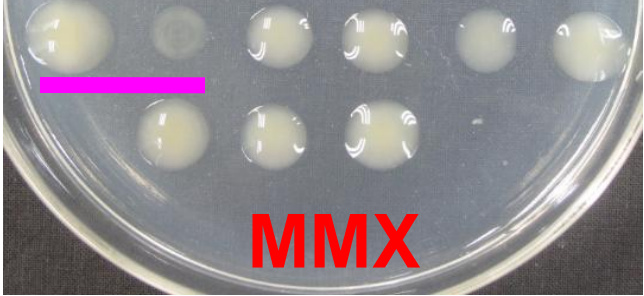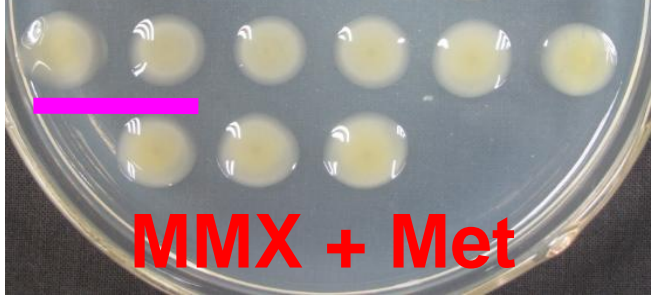

Repeat-I

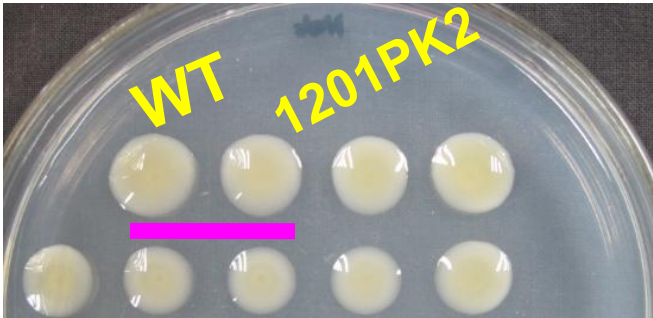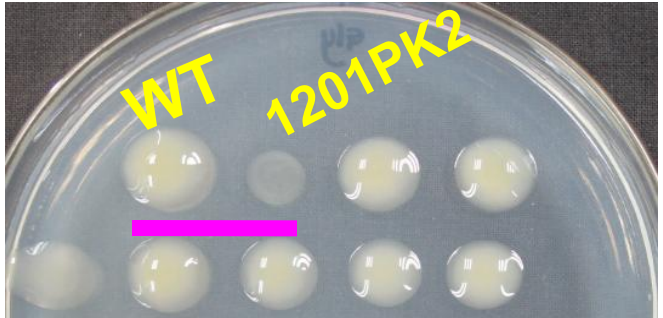

Repeat-II

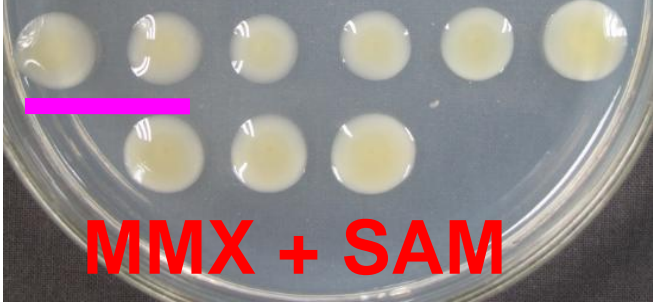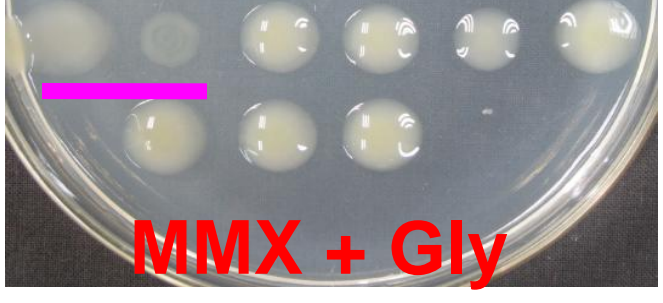

Supplement: Supplementary file 4 — Source Data [file 41467_2020_16417_MOESM4_ESM.zip › Tang et al. Source Data file/the Source Data of Supplementary Fig.3.pdf]

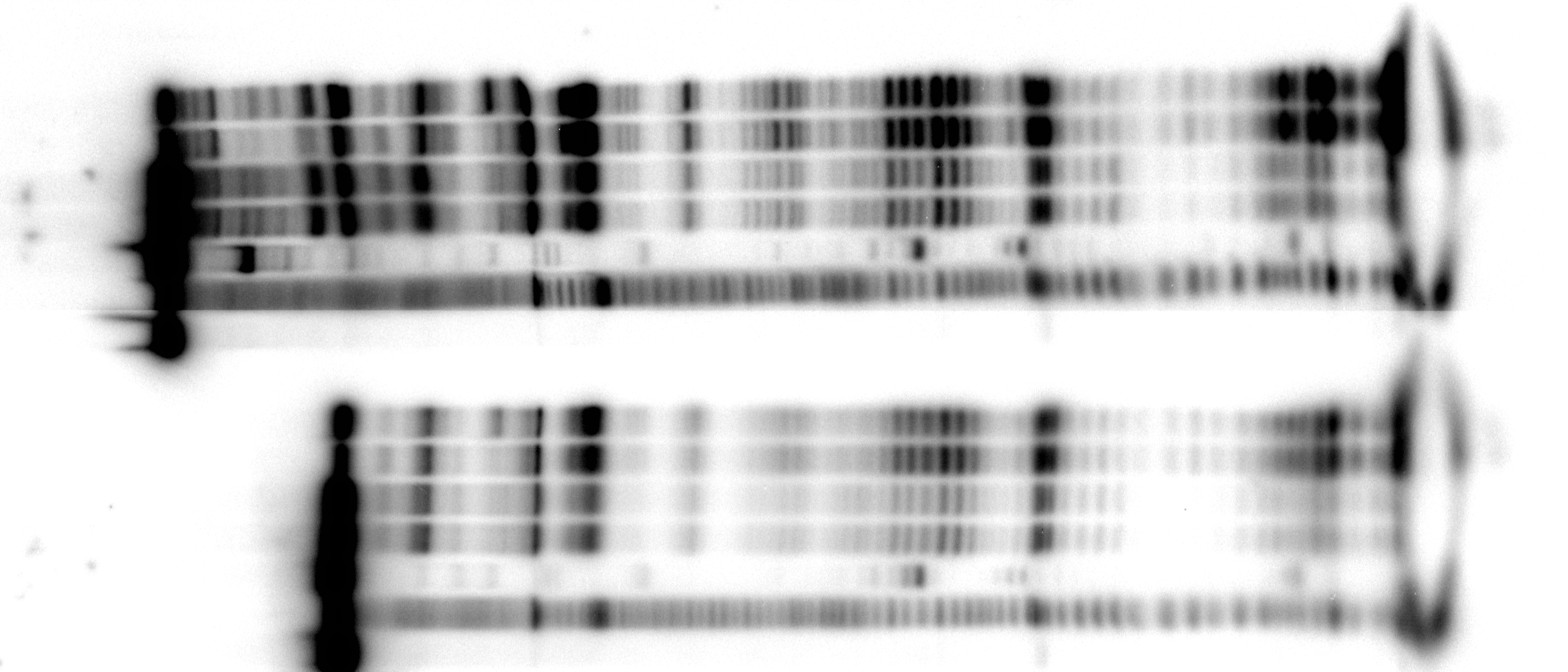

Supplement: Supplementary file 4 — Source Data [file 41467_2020_16417_MOESM4_ESM.zip › Tang et al. Source Data file/the Source Data of Supplementary Fig.5.tif]

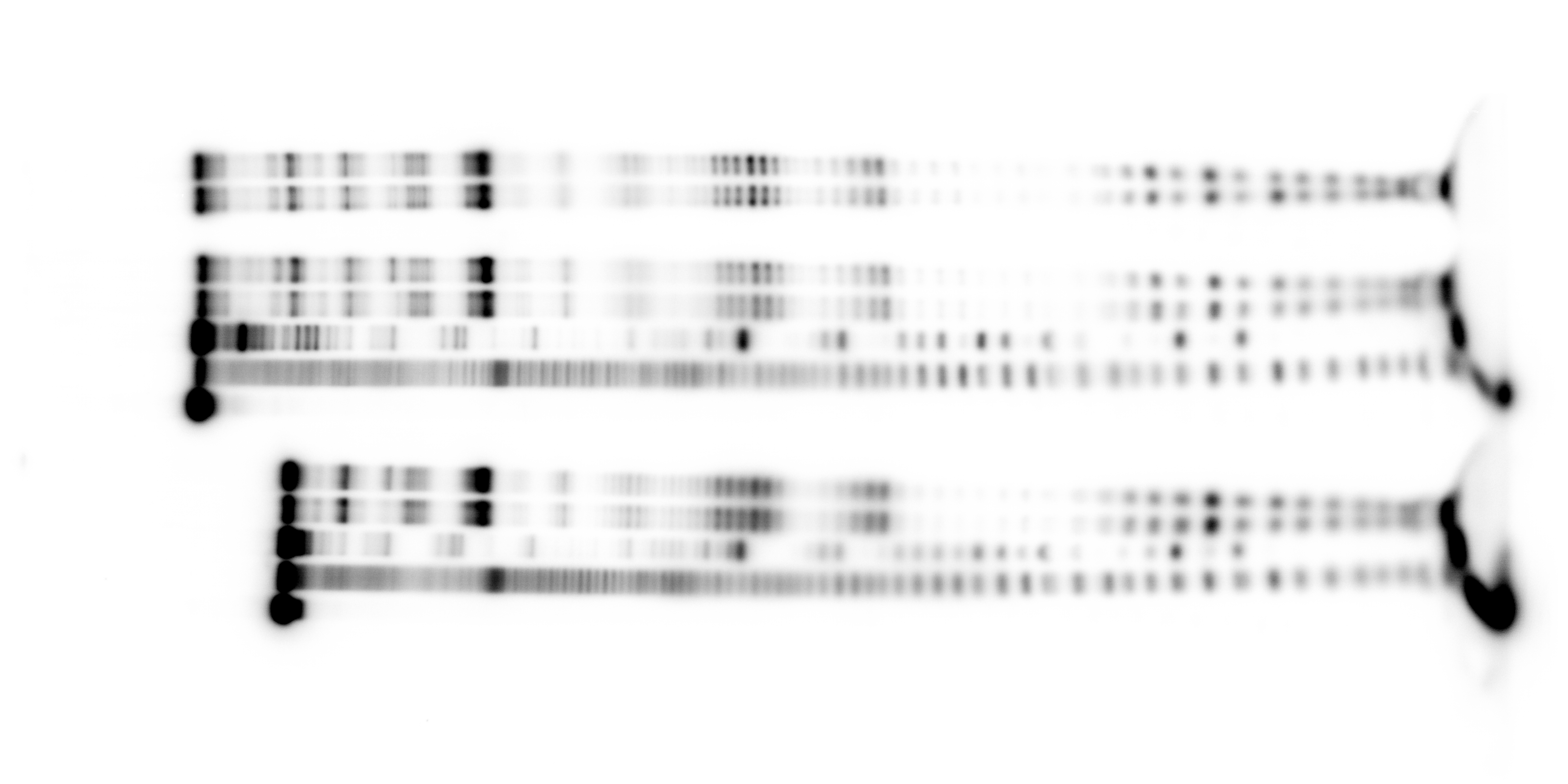

Supplement: Supplementary file 4 — Source Data [file 41467_2020_16417_MOESM4_ESM.zip › Tang et al. Source Data file/the Source Data of Supplementary Fig.6.tif]

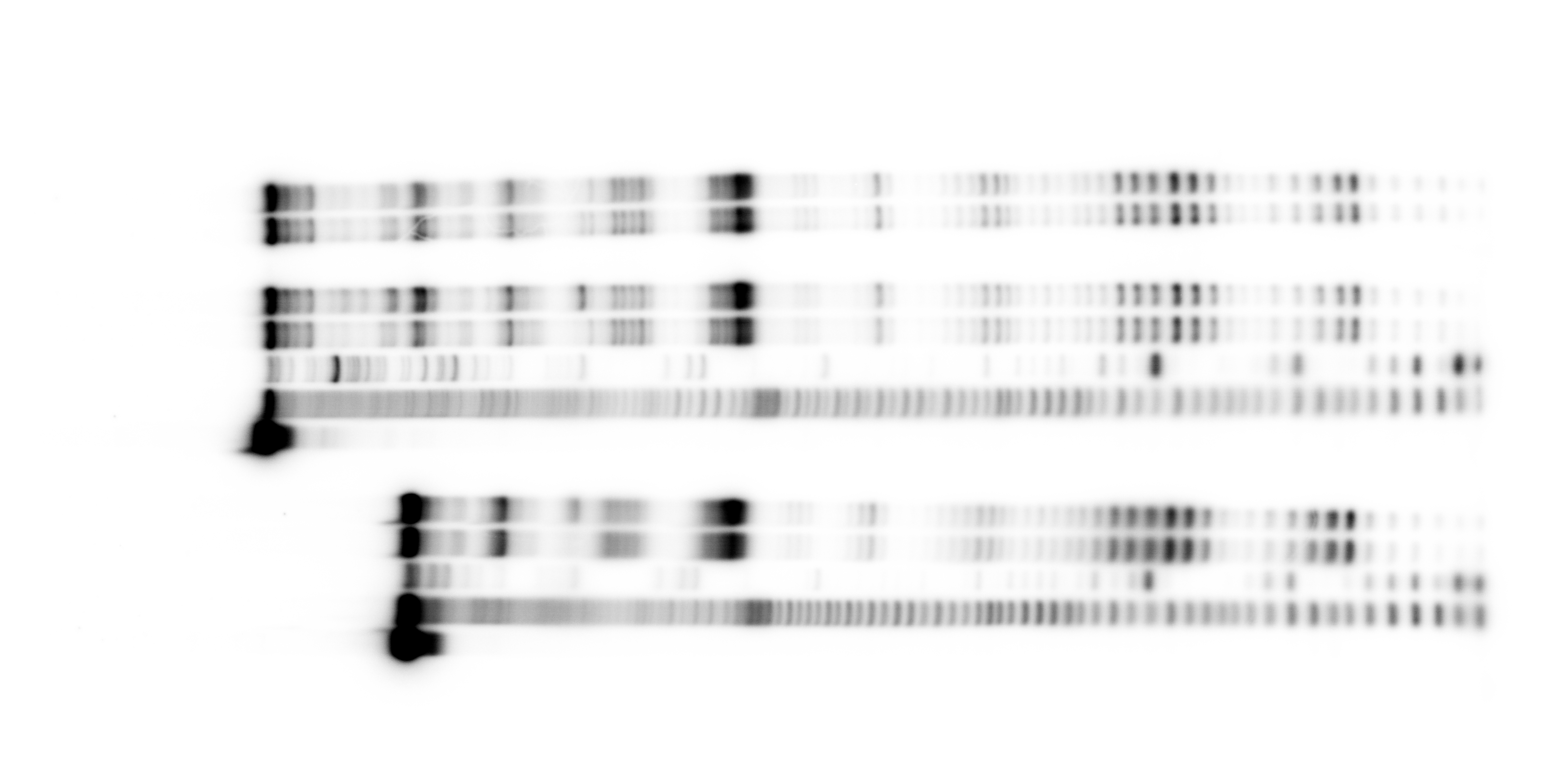

Supplement: Supplementary file 4 — Source Data [file 41467_2020_16417_MOESM4_ESM.zip › Tang et al. Source Data file/the Source Data of Supplementary Fig.7.tif]

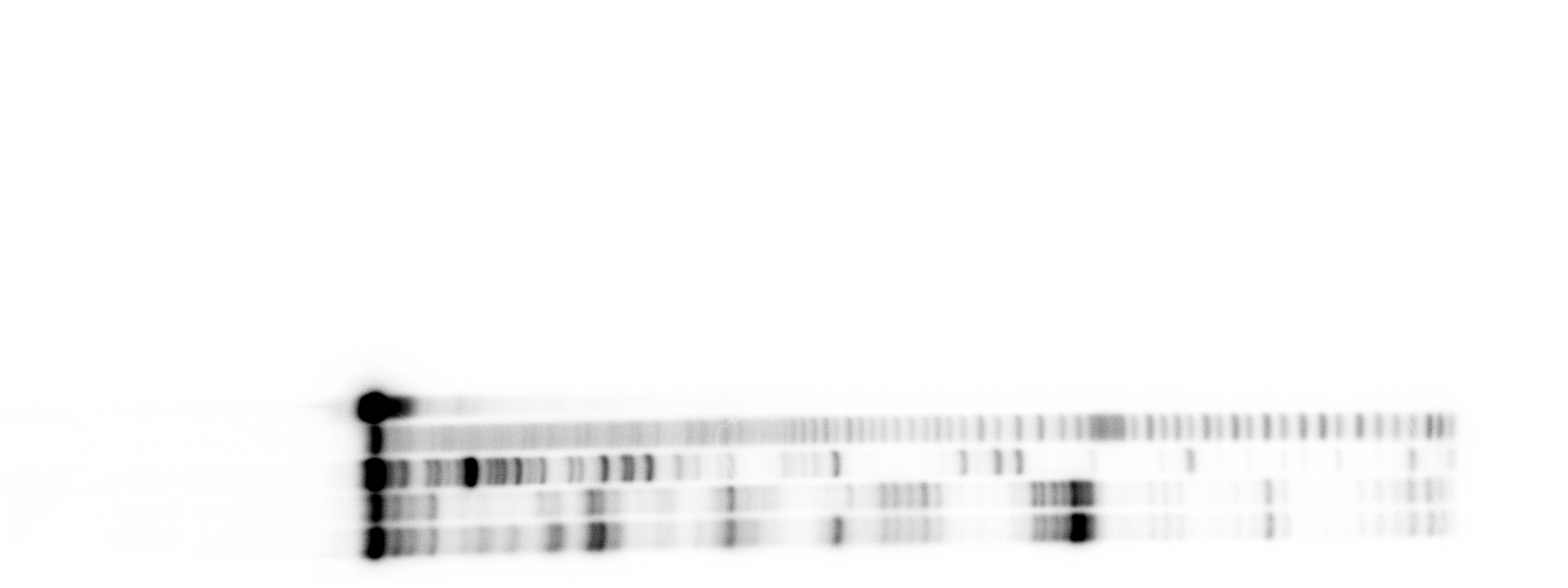

Supplement: Supplementary file 4 — Source Data [file 41467_2020_16417_MOESM4_ESM.zip › Tang et al. Source Data file/the Source Data of Supplementary Fig.8.tif]

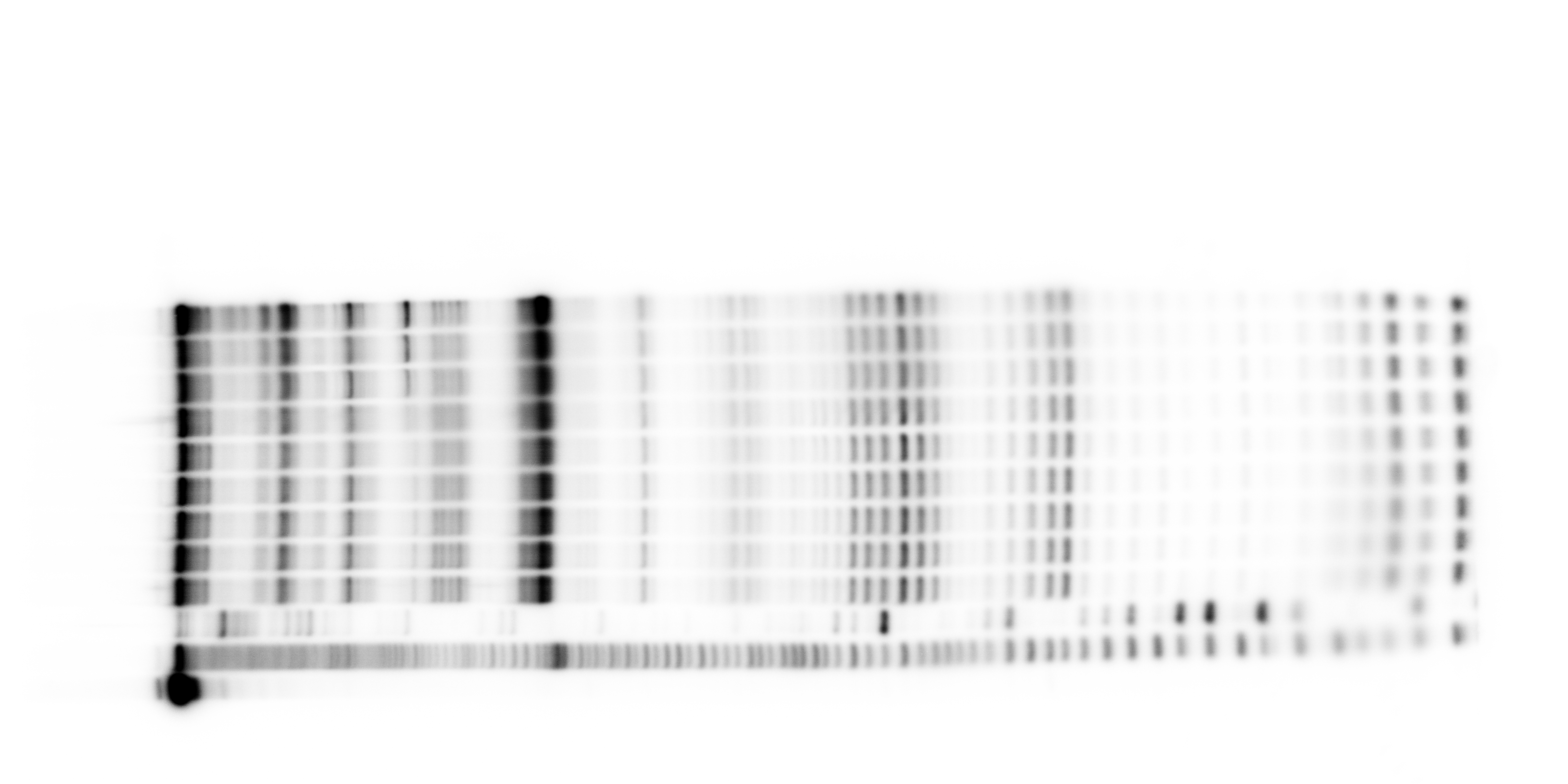

Supplement: Supplementary file 4 — Source Data [file 41467_2020_16417_MOESM4_ESM.zip › Tang et al. Source Data file/the Source Data of Supplementary Fig.9.tif]
